# Elucidating the Role of Microstructure in Thiophosphate Electrolytes – a Combined Experimental and Theoretical Study of *β*‐Li_3_PS_4_

**DOI:** 10.1002/advs.202105234

**Published:** 2022-04-24

**Authors:** Tugce Ates, Anton Neumann, Timo Danner, Arnulf Latz, Maider Zarrabeitia, Dominik Stepien, Alberto Varzi, Stefano Passerini

**Affiliations:** ^1^ Helmholtz Institute Ulm (HIU) Helmholtzstrasse 11 89081 Ulm Germany; ^2^ Karlsruhe Institute of Technology (KIT) P.O. Box 3640 76021 Karlsruhe Germany; ^3^ German Aerospace Center (DLR) Institute of Engineering Thermodynamics Pfaenwaldring 38‐40 70569 Stuttgart Germany

**Keywords:** grain boundary resistance, lithium thiophsophate, microstructure, solid electrolyte, theroretical simulation

## Abstract

Solid‐state batteries (SSBs) are promising candidates to significantly exceed the energy densities of today's state‐of‐the‐art technology, lithium‐ion batteries (LIBs). To enable this advancement, optimizing the solid electrolyte (SE) is the key. *β*‐Li_3_PS_4_ (*β*‐LPS) is the most studied member of the Li_2_S‐P_2_S_5_ family, offering promising properties for implementation in electric vehicles. In this work, the microstructure of this SE and how it influences the electrochemical performance are systematically investigated. To figure this out, four batches of *β*‐LPS electrolyte with different particle size, shape, and porosity are investigated in detail. It is found that differences in pellet porosities mostly originate from single‐particle intrinsic features and less from interparticle voids. Surprisingly, the *β*‐LPS electrolyte pellets with the highest porosity and larger particle size not only show the highest ionic conductivity (up to 0.049 mS cm^–1^ at RT), but also the most stable cycling performance in symmetrical Li cells. This behavior is traced back to the grain boundary resistance. Larger SE particles seem to be more attractive, as their grain boundary contribution is lower than that of denser pellets prepared using smaller *β*‐LPS particles.

## Graphical abstract

Overview on the relationship of particle size and pellet porosity versus ionic conductivity of *β*‐LPS thiophospate.

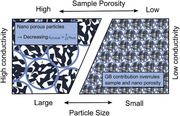

## 1 Introduction

As the interest for electric vehicles rapidly increases, new battery cell designs and chemistries need to be introduced to meet the demand of our society. The most stringent requirements for electric vehicles, thus batteries, are long driving ranges, fast charging, and safety.^[^ ^1^ ^]^ Solid‐state batteries using inorganic solid electrolytes (SEs) promise to increase energy density by enabling high‐voltage positive electrodes (cathodes) and the lithium metal negative electrode (anode). In addition, SEs may guarantee higher safety in contrast to the established LIB technology, which makes use of highly flammable organic liquid electrolytes.^[^ ^2^, ^3^ ^]^

Among different inorganic solid electrolytes, lithium‐ion conducting thiophosphates have particularly attracted the attention of the battery community. Besides the high Li^+^ conductivity, the relatively higher ductility compared to oxide‐based electrolytes makes thiophosphates particularly suitable for applications.^[^ ^4^, ^5^, ^6^, ^7^ ^]^ Li_3_PS_4_ is the most known member of the thiophosphate family, which was first described by Tachez *et al*.^[^ ^8^ ^]^ in 1984. Since then, the research target has been to understand and further improve its properties for industrial application in large‐scale batteries. One key factor for scaling‐up SSBs is the processability of electrodic and electrolytic materials. In literature, it was shown that slurry‐based manufacturing processes of SSBs consisting of Li_3_PS_4_ electrolytes is not only possible, but also very promising.^[^ ^9^, ^10^, ^11^, ^12^ ^]^ Despite this successful demonstration, one critical hurdle for the practical application of sulfide‐based SEs still remains: their instability in the ambient atmosphere. In fact, conventional phosphorus‐containing sulfides show poor stability in the air against both moisture and oxygen, which is related to the high oxygen affinity of P^5+^.^[^ ^13^ ^]^ Nevertheless, for Li_3_PS_4_ the structural changes seem to be less pronounced and, e.g., this issue can be solved, partially at least, by including additives.^[^ ^14^, ^15^, ^16^ ^]^

Li_3_PS_4_ can occur in three different phases, *α*−, *β*−, and *γ*−Li_3_PS_4_, having different ionic conductivity, depending on the arrangement of the PS_4_ tetrahedral units. The room temperature stable *γ*−Li_3_PS_4_ has a low conductivity of only 3.0×10^–7^ S cm^–1^, whereas the high‐temperature *β*−phase shows the highest ionic conductivity of 3.0×10^–2^ S cm^–1^, at 500 K.^[^ ^13^, ^17^ ^]^

In the past, different approaches have been used to synthesize highly conductive Li_3_PS_4_ by wet chemistry. The choice of solvent is very crucial, influencing the particle size and the morphology of the product due to differences in the solvent‐solid interactions during the synthesis process. For example, by employing ethyl propionate or acetonitrile, plate‐like particles were always obtained, but with rather different ionic conductivities, 2.0×10^–4^ S cm^–1^ and 7.2×10^–5^ S cm^–1^, respectively.^[^ ^18^, ^19^ ^]^

Liu et al.^[^ ^20^ ^]^ demonstrated enhanced conductivity of the *β*−phase prepared via a wet synthesis in THF. Interestingly, the introduction of nanopores resulted in the room temperature‐ionic conductivity increase of three orders of magnitude from 3×10^–7^ S cm^–1^ up to 2×10^–4^ S cm^–1^. Liu et al. claimed that since the nanoporous *β*‐LPS has a high surface‐to‐bulk ratio (rod‐shaped particles between 10 and 30 µm with an average BET specific surface area of 15.6 m^2^ g^–1^), surface conduction is dominant. Although a certain amount of porosity appears to have a beneficial effect on ion conduction, only a few papers^[^ ^21^, ^22^, ^23^ ^]^ have addressed the role of microstructure in thiophosphates, confirming the experimental results of Liu *et al*.^[^ ^20^ ^]^

In fact, while the role of microstructure and internal interfaces—such as grain boundaries (GBs, a term commonly used to describe interparticle interfaces in polycrystalline samples, as it is the case in this work)—has been extensively studied in the case of oxide SEs,^[^ ^24^, ^25^, ^26^, ^27^, ^28^ ^]^ this aspect has been largely neglected in sulfides. However, sulfide SEs can suffer from issues at GBs too, e.g., lithium tends to propagate along the GBs resulting in dendrite growth.^[^ ^29^, ^30^ ^]^ Although densification of SE powders and application of external pressure to the cell may reduce the influence of GBs,^[^ ^31^, ^32^ ^]^ these methods can only partially suppress the GB contribution, which still affects the total conductivity.^[^ ^33^, ^34^ ^]^

In this paper we give, for the first time, a comprehensive insight into the microcosmos of *β*‐LPS SEs, with the aim of correlating microstructural properties—at the particle and pellet level—with ionic conductivity and electrochemical performance. Therefore, four different batches of *β*‐LPS received from the same supplier have been systematically investigated. Despite having very similar chemical and structural composition, the four materials possess largely different particle sizes, making them an ideal set for studying the interplay between morphology and electrochemistry.

## 2 Experimental Section

### 2.1 Physicochemical and Morphological Characterization of SE Powders and Pellets

Four batches of *β*‐LPS SE were investigated as received from the supplier (BASF). In the following, the *β*‐LPS electrolytes were differentiated by numbering them as B1, B2, B3, and B4. All four batches were characterized—either in form of powder or pellet—by means of XRD, Raman, SEM, BET, SAXS, and XPS. The crystalline structure was determined via X‐ray diffraction (XRD) on a BRUKER D 8 Advance. Si‐single crystal sample holders were used with a cap and knife to protect the sample from air contact. Raman spectroscopy measurements were performed on a Renishaw with a 633 nm confocal red laser source and 50× objective lens configuration. The pristine particles were morphologically characterized via scanning electron microscopy with a ZEISS Gemini Field Emission SEM using a primary electron beam of 3 kV. All samples under test were fixed onto an aluminum sample holder with a conductive carbon tape (Plano G3347), and placed in a transport box to prevent contamination with air. The cross‐sectional morphology of the pellets was investigated by SEM after groving them with a focused ion beam (FIB, sputtering with Ga ions for almost 6 h). The BET surface area of the powders was obtained by N_2_ desorption isotherms measured with a Quadrosorb‐iQ, Quantachrome Instrument. For the SAXS and XPS description, see Supporting Information.

All samples were prepared, handled, and stored under Argon atmosphere inside a glovebox. To prevent contamination, they were transported using air‐tight vessels.

### 2.2 Electrochemical Characterization

#### 2.2.1 Pellet Preparation

The SE pellets were prepared by compressing 150 mg of the various SE powders inside a pressing mould with a diameter of 10 mm. It is important to mention that the thickness of the electrolyte pellets could not be fixed in the following experiments, and only the amount of SE powder was kept the same, to maintain the results comparable. As the densification depends on the microstructure, the pellets show slightly different thicknesses correlating with their degree of porosity. The thicknesses were determined by the means of a micrometer. As the fabrication and operating stack pressure was also crucial for SSBs,^[^ ^35^, ^36^ ^]^ the pellet fabrication pressure was fixed at 300 MPa for 30 s at room temperature for all samples.

#### 2.2.2 Cell Assembly

For the electrochemical measurements, two‐electrode torque cells for the assembly of pellet‐type solid cells (Wellcos cells) were used. The electrolyte pellets were sandwiched between two stainless steel for ionic conductivity measurements via electrochemical impedance spectroscopy (EIS) or between two lithium electrodes for additional impedance measurements and stripping/plating tests. The operating stack pressure was fixed at 5 Nm (≅ 0.04 MPa) for all cells. The cells were placed in a climatic chamber (Binder) for temperature control during all measurements.

The ionic conductivity of the pellets from the four *β*‐LPS batches, as well as their stablitiy toward the lithium metal electrode, was investigated by electrochemical impedance analysis (EIS; by a Solartron Analytical (Ametek) impedance analyzer 1260). A 10 mV sinusoidal amplitude was applied in the 1 MHz to 1 Hz frequency range. The ionic conductivity of the electrolytes was measured from 20 to 90 °C with 10 °C steps. Before each measurement, the cells were allowed to thermally stabilize for 2 h.

The stability of the Li/*β*‐LPS interface was evaluated by monitoring the time dependence of the impedance response of symmetric Li/*β*‐LPS/Li cells kept at 20 °C under open‐circuit voltage (OCV) conditions.

The suitable fitting of the EIS measurements, performed by using a proper equivalent model circuit, allowed to evaluate the contribution of the bulk and interfacial resistances to the overall cell impedance, and to monitor their change as a function of time. All fits were carried out with the RELAXIS software (rhd Instruments).

Lithium stripping/plating tests were performed in symmetric Li/*β*‐LPS/Li cells held at 20 °C, using a VMP ‐3 potentiostat/galvanostat. Galvanostatic cycles were run by applying 0.1 mA cm^–^^2^ for 1 h each step.

### 2.3 Simulation Workflow

In this work, microstructure resolved simulations were performed to account for the influence of grain and grain boundary structures on lithium transport in the SE. The simulation approach enabled us to resolve the time and space‐dependent concentration and potential evolution for different electrochemical cell setups. For a detailed model description, refer to Refs. ^[^ ^37^, ^38^, ^39^, ^40^ ^]^ and details on the simulation workflow and model equations are also given in the Supporting Information.

#### 2.3.1 Generation of Virtual SE Pellets

**Figure 1:**
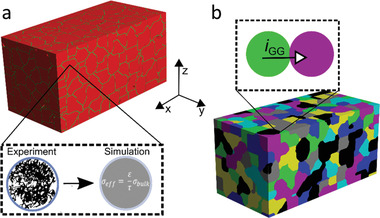
a) Exemplary image of a segmented SE microstructure with 20 µm particle size. b) As shown in the inset, we specify single particles in the segmented structure to apply the GB interface model at adjoint grains. The color code is used as a guide for the eye, and despite the different colors, all grains do have the same physical and chemical properties.

Virtual SE pellets for the simulation study were generated in GeoDict^[^ ^20^ ^]^ assuming convex polyhedral particles following a Gaussian distribution of the particle diameter (see Figure S6, Supporting Information). The digital twins of the resulting SE pellets had a voxel size of 0.5 µm to provide a reasonable resolution of SE particles. The lateral dimension of the simulation domain was set to 100 × 100 voxels with nonperiodic boundary conditions, while the SE thickness was adjusted to reproduce the dimensions of corresponding experiments (cf. **Table** **1**). Subsequently, a segmentation step to assign individual grains was performed, which allowed introducing GBs and the corresponding resistance across the interface. **Figure** **1**b shows a representative microstructure after segmentation, where the different colors represent individual grains. Despite the different colors, all grains do have the same physical and chemical properties. Further details on the transport model including GBs were given in the supporting information and Ref. ^[^ ^36^ ^]^.

**Table 1:** Calculation of the geometric density and porosity of the electrolyte pellets

	Amount [mg]	Area [cm^2^]	Thickness [cm]	Geometric density [g cm^−^^3^]	Porosity [%]
**B1**	200	1.32	0.115	1.318	≈29.5%
**B2**	200	1.32	0.135	1.122	≈39.9%
**B3**	200	1.32	0.087	1.783	≈4.5%
**B4**	200	1.32	0.090	1.684	≈9.8%

#### 2.3.2 Electrochemical Simulations

To calculate the effective sample conductivity, a constant current was applied to the virtual cell and solved for the stationary potential distribution. The current was directly applied as a boundary condition at the SE pellet neglecting the electrode and charge transfer resistance. Additionally, impedance simulations in a blocking electrode configuration were performed. Details on the impedance setup and simulations are provided in the Supporting Information for additional validation of the theoretical study.

### 2.4 Statistical Analysis

Statistical analysis was necessary to approximate the particle sizes of the different batches and their respective ionic conductivities. The determined particle sizes refer to the secondary particles estimated from the SEM images collected at lower magnification reported in Figure 3. To estimate the particle sizes, Image J was used. The resulting data were presented as mean ± SD (standard deviation), whereas an average of 10 particles was considered for each batch. The ionic conductivities shown in the manuscript were the average values of four measurements and were also presented as mean ± SD.

## 3 Results and Discussion

### 3.1 Particle Characterization

The pristine electrolyte powders were characterized in terms of crystallinity, purity, morphology, surface area, and surface chemistry by XRD, Raman, XPS, SEM, SAXS, and BET measurements.

**Figure 2:**
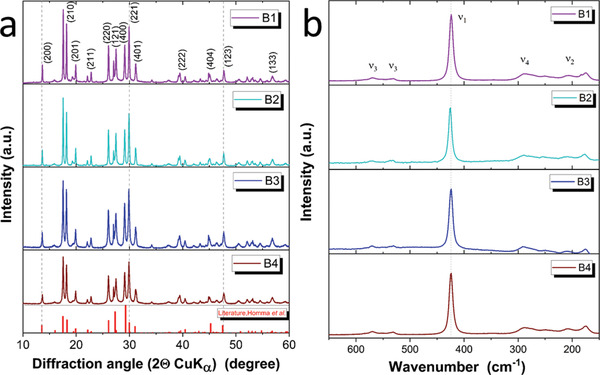
a) XRD pattern and b) Raman spectra of the different batches of *β*‐LPS batches. From top to bottom: In violet: B1, in turquoise: B2, in dark blue: B3, and in dark red: B4.

The XRD patterns and Raman spectra of the SE batches B1–B4 are shown in **Figure** **2**a,b, respectively. The XRD patterns do not differ within the four batches, fitting very well with the literature results.^[^ ^8^, ^17^, ^20^ ^]^ The theoretical density of the four samples and their lattice parameters were calculated from the XRD patterns (see Table S1, Supporting Information). Once more, all the calculated values are very similar and consistent with the literature data.^[^ ^17^ ^]^ Additionally, the average crystallite size (D) was estimated via the Scherrer equation (1)^[^ ^41^ ^]^:

(1) $$D=\frac{K*\lambda }{\beta *\cos\left(\theta \right)}$$

where *K* = shape factor (0.9), *λ* = X‐ray wavelength, *β* = FWHM, and *θ* = Bragg angle.

The average crystallite (or grain) size of B1 and B2 are in the same range, 37.3 and 37.1 nm, respectively, whereas the other two batches have ≈30% smaller crystallite sizes, showing values of 27.0 nm for B3 and 25.8 nm for B4.

The acquired Raman spectra show that the four electrolyte powders do not contain major, Raman active, bulk impurities. The only observed functional group is the PS_4_^3–^ tetrahedron, showing the characteristic symmetric stretching (*ν*_1_) at ≈425 cm^–1^. The less intense peaks at lower wavelengths, i.e., 279 and 214 cm^–1^ represent, respectively, the asymmetric bending (*ν*_4_) and the symmetric bending (*ν*_2_) of the same group. At higher wavelengths, the peak of asymmetric stretch can be observed at 535 cm^–1^ (*ν*_3_).^[^ ^8^, ^20^, ^42^ ^]^ Overall, in terms of bulk properties of the powders, it can be concluded that there is no evident difference between the four batches besides their average crystallite size.

Although no impurities could be detected by XRD and Raman, the XPS investigation revealed some subtle differences in terms of surface chemistry, as discussed in detail in the Supporting Information (see Figure S1, Supporting Information). Interestingly, LiOH was detected on B2 and B3 particles. Additionally, these two batches show the presence of H_2_S. These impurities, however, were not detected in B1 and B4.

**Figure 3:**
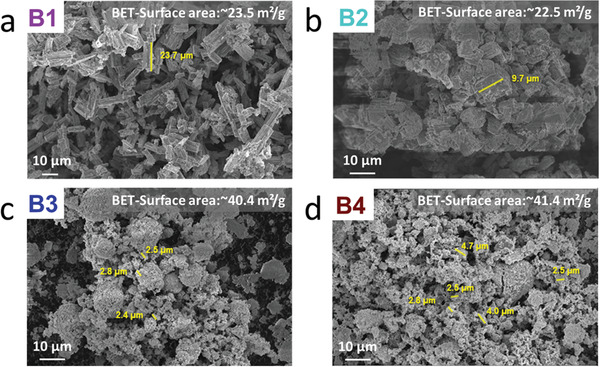
SEM images and BET‐surface area of a) B1, b) B2, c) B3, and d) B4.

Looking closer at the morphology of the particles displayed in the low magnification SEM images in **Figure** **3**, obvious differences between the four SE batches arise. The first two batches (B1 and B2) show the characteristic rod‐shaped particles with a rough surface morphology, similar to those observed by Liu et al. and others.^[^ ^20^, ^21^ ^]^ On the contrary, B3 and B4 particles have no clearly defined shape. Additionally, these particles are substantially smaller. In fact, they have average lengths of 3.71 ± 0.89 µm (B3) and 5.53 ± 1.14 µm (B4), while B1 and B2 display (on average) mean particle lengths of 20.69 ± 3.81 µm and 6.90 ± 1.67 µm, respectively. The values for the particle size refer to the secondary particles estimated from the SEM images reported in Figure 3. Additionally, some SEM images with high magnification are reported in Figure S2 (Supporting Information). For simplification, the particle size will be approximated in the following text as: B1 – 20 µm, B2 – 7 µm, B3 and B4 each 3 µm.

The different particle size is reflected onto the surface area of the SEs, as confirmed by the nitrogen adsorption measurements and BET analysis (whose values are given in Figure 3). The first two batches having larger particles show similar BET values, slightly higher than 20 m^2^ g^–1^. On the other hand, the SE batches B3 and B4 display BET‐surface areas ≈40 m^2^ g^–1^. From the rather rare reports dealing with the surface area of LPS electrolyte powders, values ranging between 15.6 m^2^ g^–1[^ ^20^ ^]^ and 24.0 m^2^ g^–1[^ ^33^ ^]^ are found. The large surface area of B3 and B4 is, therefore, quite unconventional. Further, the determined values seem to be below what is actually theoretically expected for these two batches according to their particle size. The overall surface area of B3 and B4 might be even higher, but due to agglomerated particles, as seen in Figure 3 and Figure S2 (Supporting Information), the internal pores may be poorly accessible.

### 3.2 Pellet Characterization

In order to conduct electrochemical measurements, the SE powders were densified into pellets.

In Table 1, the calculated geometric densities and porosities of the pelletized SEs are listed. It is clear that for B1 and B2 large voids remain in the pellets according to the measured 29.5% and 39.9% porosity, respectively. The porosity of B3 and B4 pellets is considerably lower, with values of “only” 4.5% and 9.8%, respectively. The pellet density seems determined by the intrinsic porosity of the secondary particles. In fact, it is not possible to further reduce the porosity of the B1 and B2 samples by increasing the applied pressure. Although sulfides are known to be ductile, they become glassy and brittle when densified.^[^ ^12^, ^43^, ^44^, ^45^ ^]^ An increase in pressure resulted in broken pellets, especially for the highly porous samples. Therefore, the applied pressure was maintained identical for all samples, to show that at given conditions the particle morphology has an impact on the pellet porosity.

**Figure 4:**
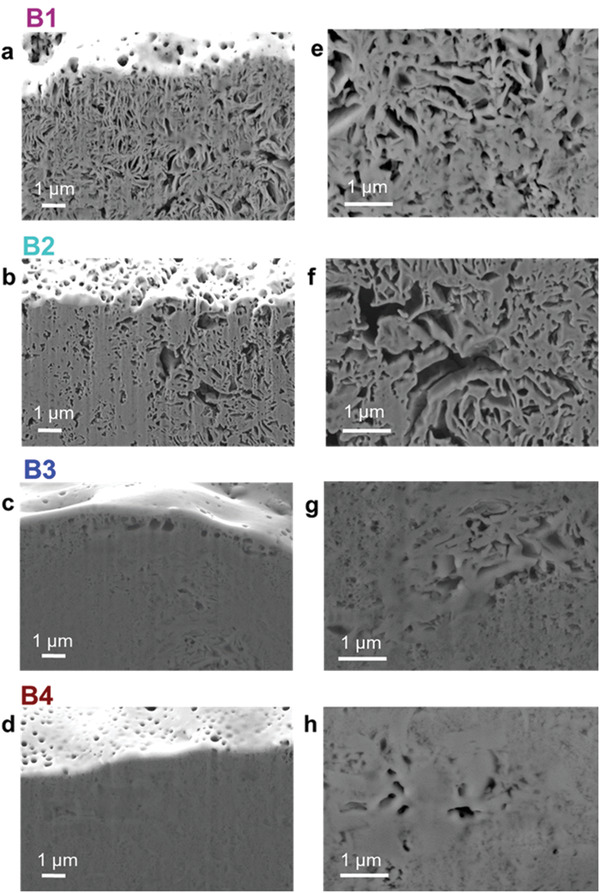
Cross‐section images of the SE pellets were obtained by SEM‐FIB. B1 in a) low and e) high magnification, of B2 in b) low and f) high magnification, of B3 in c) low and g) high magnification and of B4 in d) low and h) high magnification.

The porous character of the pellets could be confirmed by SEM cross‐sections obtained by FIB. In **Figure** **4**, the cross‐sectional images at low (Figure 4a–d) and high (Figure 4e–h) magnifications are shown. It is evident that B1 and B2 (Figure 4a,e and Figure 4b,f) possess large pores reminding a sponge‐like structure. Differently, B3 and B4 appear evidently denser, also due to the smaller particles being easier to compact.

With the aim of reducing the large porosity of B1 without damaging the pellet, the pressing procedure was performed under an active vacuum too. Although, no further densification of the SE could be achieved (see Figure S3a,b, Supporting Information). This again suggests that the large porosity of B1 and B2 is an intrinsic feature of the large secondary particles, and does not arise from voids between adjacent particles. This was confirmed by performing a cross‐section of a single *β*‐LPS particle from B1 (see Figure S3c–e, Supporting Information), from which pores within a single particle are seen between single crystallites as also described by Liu et al.^[^ ^20^ ^]^ Such an intrinsic particle porosity is, apparently, not removable by cold‐pressing.

SAXS measurements were also performed to gain information about pore shape and average diameter. The SAXS measurements are displayed in Figure S4 (Supporting Information). From the slope of the curves in a log‐log plot, oblate spherical pore‐shapes can be proposed for B1 (slope: −3.5) and B2 (slope: −3.7). Based on the radius of gyration, the distribution average pore size was determined to be 20 nm for B1 and 50 nm for B2. Whereas, for B3 and B4 two different pore shapes can be proposed, oblate spheres (slope: −3.8) and disks (slope: −1.7 and −1.8), with an average pore size of 18 nm and 15 nm, respectively. Liu et al. reported an average pore diameter of 28 nm determined by SAXS, too.^[^ ^20^ ^]^ Herein, it is assumed that the oblate spherical pores originate from those existing in the powder particles, which still remain after pelletizing. The additional disk‐shaped pores detected for B3 and B4 might be voids occurring between particles. Nevertheless, a polydisperse distribution applies for all four batches (Figure S4, Supporting Information), which explains the discrepancy with the porosity observed in the SEM images.^[^ ^20^, ^46^, ^47^ ^]^

**Figure 5:**
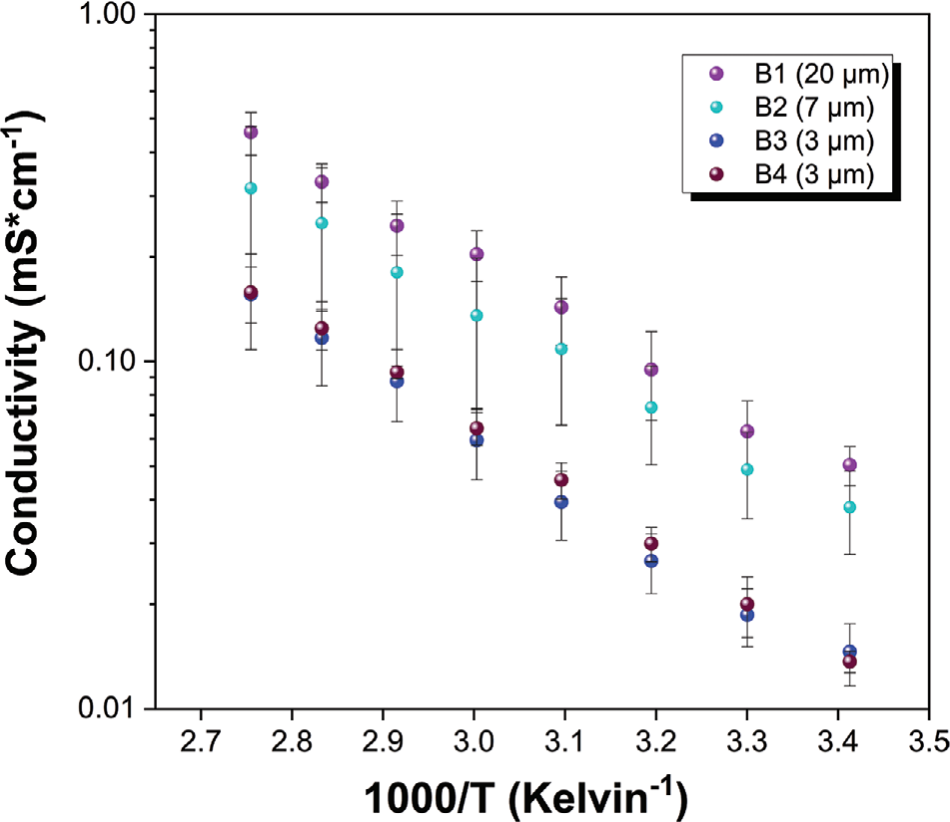
Ionic conductivity in symmetrical cells with stainless steel blocking electrodes and the respective statistical error given as error bars. In violet: B1, in turquoise: B2, in dark blue: B3, and in dark red: B4.

The ionic conductivities of the four *β*‐LPS samples were measured by EIS and are reported in **Figure** **5**. The representative Nyquist plots, the equivalent circuits, and the equation to calculate the ionic conductivities are shown and described in the supplementary information (Figure S8, Supporting Information). The highest ionic conductivity is provided by B1 (4.9×10^–5^ ± 0.6×10^–5^ S cm^–1^ at 20 °C), followed by B2 (3.7×10^–5^ ± 1.7×10^–5^ S cm^–1^ at 20 °C) and B3 (1.6×10^–5^ ± 0.2×10^–5^ S cm^–1^ at 20 °C), while B4 shows the lowest value (1.5×10^–5^ ± 0.6×10^–5^ S cm^–1^ at 20 °C). The relatively low ionic conductivities of these LPS batches compared to previous literature may be due to their nanocrystallinity. In fact, partially amorphous samples commonly show higher conductivities.^[^ ^20^ ^]^ However, besides the absolute conductivity values, the observed trend is quite surprising, with the highly porous pellets formed by B1 and B2 allowing faster Li^+^ transport than the denser B3 and B4 pellets. The activation energies were also determined by using the Arrhenius equation Equation S7 (Supporting Information), and are reported in Table S7 (Supporting Information). All four batches show values in the same range: 0.29–0.33 eV.

### 3.3 Simulation Results

In order to exhaustively explain the reason behind the different ionic conductivity of these SEs, a microstructure resolved simulation study was performed to investigate the dependency of the ionic conductivity on porosity, tortuosity, and GB contributions.

In the simulation workflow, digital SE structures are generated with three different representative particle diameters. This allows to correlate the microstructural properties with the effective conductivity. The particle sizes for the three virtual SE samples are set to 20 µm, 7 µm, and 3 µm. The resulting microstructures are shown in the supporting information (Figure S6, Supporting Information).

The cross‐sectional SEM images show a considerable internal porosity of SE particles. Based on the material density porosities between 10% and 40% are estimated as reported in Table 1. In the simulations, a homogenization approach is used to describe the Li^+^ transport within the porous particles (c.f. Figure S6, Supporting Information). It should be noted that Equation 2 allows qualitative assessment of the effect of internal particle microstructure and significantly reduces the computational cost. The effective particle bulk conductivity^[49]^ is given by

(2) $$\sigma_{\mathrm{eff},\;\mathrm{bulk}}=\frac{\varepsilon }{\tau^{2}}\sigma_{\mathrm{bulk}}$$

where *ε* is the particle porosity and *τ* the tortuosity, as shown in the inset of Figure 1a. The bulk conductivity value is set to *σ*_*bulk*_ =  2.2  ×  10^−4^ S cm^–1^ based on NMR measurements, accounting for the short‐range Li‐ion mobility.^[^ ^33^ ^]^ Therefore, the bulk conductivity value excludes long‐range hopping processes related to GB transport. Since the determination of the internal tortuosity is not straightforward we perform parameter variations with tortuosity values between 1.3 to 2.0. This range was chosen based on previous studies on porous oxide‐based SEs.^[^ ^24^ ^]^ Variation of the respective structural parameters in Equation 2 allows a qualitative assessment of the effect of internal particle constriction on the transport resistance. An overview of the resulting effective bulk conductivity of the particles for the given combination of tortuosity and porosity is given in Table S2 (Supporting Information). Note that GBs have a significant contribution to the resistance of the pellets, decreasing the effective conductivity (*σ*_eff_). These contributions are accounted through the GB interface model, as depicted in Figure 1b. Thus, the effective conductivity (*σ*_eff_) is affected by i) the particle‐related effective bulk resistance and ii) the superimposing microstructure and GB related interface resistances. The latter depends on the particle size since for small particles the number of GBs in the same volume increases. Since the effective conductivity calculations are done using steady‐state simulations under constant current conditions on the complex geometry, a general decomposition of both contributions is not straightforward. Nevertheless, additional information on the general impedance response and the comparison between experimental and simulated impedance data is provided in Figure S11 (Supporting Information).

### 3.4 Effective Sample Conductivity

**Figure 6:**
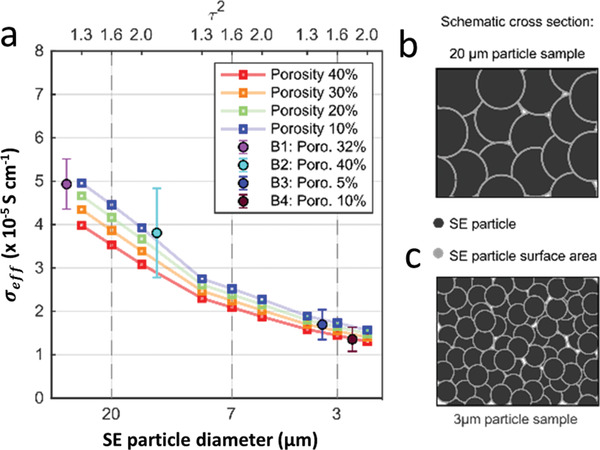
a) Calculated effective SE conductivities for three different microstructures with varying particle diameter. The continuous lines connect values with the same porosity, and the open circles represent the respective tortuosity (top axis). The filled circles give the experimental conductivity values obtained with stainless steel blocking electrodes, b) schematic cross‐section of 20 µm particle samples, and c) schematic cross‐section of 3 µm particle samples.

The calculated effective conductivities for the three structures with changing SE particle diameter are given in **Figure** **6**. Each line represents a fixed porosity, and the three open symbols represent tortuosity values of 1.3, 1.6, and 2.0, respectively. Full circles represent experimentally obtained values and their respective statistical errors are given as error bars.

Calculated effective conductivity values are in the range between 1.2 and  5 · 10^−4^ S cm^–1^, i.e., the deviation of the experimental data is given by the statistical spread, which cannot be simulated. More importantly, the simulations reproduce the trend that is observed experimentally. The theoretical and experimental results validate the trend of decreasing effective ionic conductivity with reduced particle size and an increased GB contribution. A detailed comparison and validation study on the experimental and simulated impedance results for the respective LPS batches is additionally shown in the supporting information (cf. Figure S11 and Tables S5 and S6, Supporting Information). Interestingly, the SE particle size has a stronger impact on the conductivity than the pellet's porosity and tortuosity. Although the pellet with a 20 µm particle size has a porosity of up to 32%, the effective conductivity is by a factor of 4 higher than for the sample with 3 µm particle diameter and 10% porosity. The predicted decrease in conductivity with particle size agrees with the experiments, where B3 and B4 have the smallest particle size. This indicates that the interface resistance between grains is a dominant factor for lithium transport in LPS pellets. In order to prove this, a particle size reduction study of sample B1 was conducted. Namely, the B1 powders were ground in two different ways, manual grinding (MG) and softball milling (SB), to reduce the particle size. In both cases, a slightly reduced ionic conductivity compared to their pristine samples was observed (see Figure S5, Supporting Information).

The growing impact of the GB resistance compared to the internal particle constriction is also evident by the lower sensitivity of the effective conductivity toward changes in particle porosity and tortuosity in Figure 6a. Due to the lower surface to volume ratio of larger SE particles, the impact of the effective bulk conductivity is larger compared to the smaller particles, where this ratio increases. This observation is further supported by comparing the particle‐specific surface areas given in Table S9 (Supporting Information). The smaller SE particle diameter increases the particle specific area by a factor of 2 (7 µm) and 4 (3 µm), respectively. Figure 6b,c shows the schematic increase in particle surface area with decreasing particle size for the two complementary SE samples. This increase in the specific surface area leads to a higher contribution of the interface resistance since smaller particles effectively decrease the conductivity in the SE pellet. In addition, the smoother surface area of the larger SE particles might improve the transport on the particle surface and reduce the contact resistance.^[^ ^22^ ^]^ A detailed impedance simulation study for each LPS batch is additionally shown in Supporting Information (cf. Figure S11, Supporting Information).

The trend between the highly porous (B1+B2) and less porous (B3+B4) samples is proven experimentally and in theoretical simulations. However, among the samples with similar porosities, some changes in conductivity are observed. Besides the GB resistance, additionally, the surface impurity of the samples might play a minor role. The samples B2 and B3 feature LiOH and H_2_S surface impurities, as previously shown in Figure S1 (Supporting Information). These impurities may have an additional impact on the Li^+^ transport and explain the slightly decreased ionic conductivities for B2 and B3 compared to their “purer counterparts” B1 and B4, respectively.

In this dataset of LPS batches, it can be concluded that the grain–grain interfacial resistance definitively dominates the transport. These results emphasize that a high pellet porosity does not necessarily lead to a low ionic conductivity since the SE particle micro‐and nanostructure must be considered. Furthermore, one can assume that on a lower scale, the crystallite size might also affect the effective particle conductivity since the nano‐crystallinity changes for the batches as indicated by SAXS measurements. Similar to a larger particle tortuosity, smaller crystallites commonly decrease the effective particle conductivity. This might explain the somehow smaller experimental conductivities of B3 and B4 compared to the simulations, as discussed further in Supporting Information (Figure S12, Supporting Information). Still, the simulations favorably reproduce qualitative trends observed in the experiments providing a sound explanation for the effect of particle microstructure and GB resistance on the SE conductivity.

Overall, if the most dominant contribution to the total resistance is associated with GBs, one could expect the highest conductivity to be achievable with single‐crystal materials. In fact, single‐crystal sulfides such as Li_10_GeP_2_S_12_ reported by Iwasaki et al. demonstrated remarkably high ionic conductivities at room temperature in the range of 27 and 7 mS cm^−1^ depending on the crystal direction. Nevertheless, this kind of approach, as growing single crystal SEs is far from an easy and practical application.^[^ ^48^ ^]^

### 3.5 Half Cell Analysis

**Figure 7:**
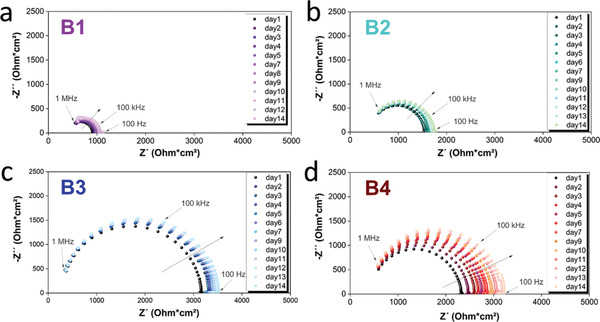
Nyquist plots of 14 days of aging of a) B1, b) B2, c) B3, and d) B4.

Finally, the electrochemical properties of the four *β*‐LPS batches were evaluated in Li/ *β*‐LPS/Li symmetric cells. To analyze the SEs’ stability against Li, the cells were stored for 14 days at OCV and 20 °C. Impedance spectra were recorded every 24h to monitor the evolution of the interfacial resistance. The Nyquist plots of all cells are plotted in **Figure** **7**.

All batches appear relatively stable against Li, as evidenced by the interfacial resistance increasing only slightly upon storage time. The overall impedance increased from 1065 Ohm cm^2^ on day 1 to ≈1482 Ohm cm^2^ on day 14 for B1, from 1539 to 1772 Ohm cm^2^ for B2, from 3147 to 3505 Ohm cm^2^ for B3, and from 1884 to 2541 Ohm cm^2^ for B4. Such impedance buildup with time indicates the formation and growth of a resistive interlayer at the Li/SE interface.

**Figure 8:**
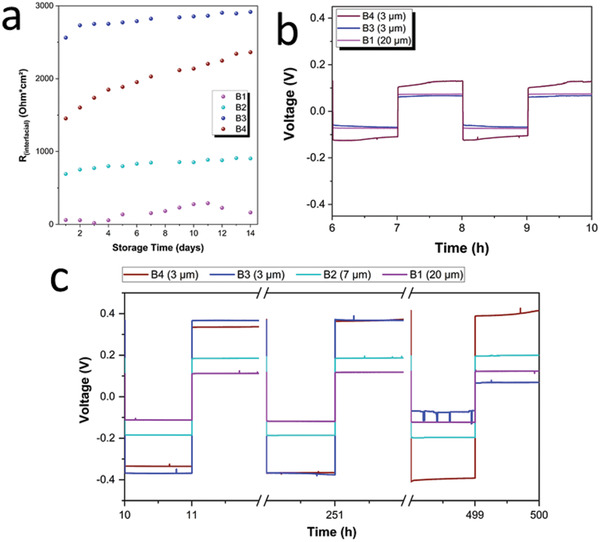
a) Evolution of the total resistance *R*_total_ upon storage time. Cell voltage evolution upon stripping/plating cycles of b) fresh cells and c) aged cells (B3 shorting after 380 h). In violet: B1, in turquoise: B2, in dark blue: B3, and in dark red: B4.

The electrolyte with the highest conductivity (B1) also shows the lowest initial interfacial resistance (and vice versa). The change in total resistance over time is also visualized in **Figure** **8**a. Here, the same trend as for the ionic conductivity plot is observed. B1, with the highest porosity and lowest grain boundary contribution, starts with the lowest overall resistance (*R*_total_) and the slowest increase upon storage time, followed by B2. In contrast, the denser B4 and B3 pellets show higher total resistances, which also grow faster upon storage time. The fast resistance growth could be related to the dense pellets having larger contact areas with the Li metal foil, leading to more extensive electrolyte decomposition. It should be also mentioned that the nature of the formed interface may be affected by impurities on the Li metal and SE surfaces. Since the same type of Li foil was used for all the cells, the influence of the passivation layer on the Li metal can be neglected. However, also no direct correlation between the interfacial resistance and, e.g., the presence of LiOH on the SE particles' surface (see XPS results in SI) can be appreciated.

Figure 8b shows the stripping/plating results of cells cycled immediately after assembling (fresh cells not subject to the 14 days of aging), while in Figure 8c the behavior of aged cells for 500 h is plotted, respectively. By comparing the overvoltage of fresh and aged cells, an increase by a factor of at least 2 can be noticed. This is due to the increased interfacial resistance during the aging test shown in Figure 8a.

Nevertheless, the trend of cell polarisation among the four samples agrees very well with the EIS measurements. B1 shows the lowest cell polarisation followed by B2, B4, and B3, which shows the highest overvoltage. For the cells aged before the stripping/plating test, the cycling performance was enhanced due to the interfacial layer already being formed. All four batches show very stable stripping/plating behavior. For the highly porous electrolytes, B1 and B2, the overvoltage remains stable even after 500 h of cycling. On the other hand, the dense pellets of B4 display an increasing overvoltage with time, and B3 suffers from a short circuit after only 380 h.

Although good cyclability for up to 500 h could be achieved for the high porous samples, one should have in mind that excessive porosity is an issue and will allow Li dendrite propagation when increasing the applied current density. Nevertheless, assuming that the issue of dendrite growth is solved differently, partially porous SE layers could help reduce the weight of this inert component in the cell. In any case, they will need to be processed into thin separator films, e.g, by tape casting. In our previous work^[^ ^9^ ^]^ we were indeed able to produce relatively thin and homogeneous separators featuring the large particle‐size, rod‐shaped Li_3_PS_4_ SE (≈20 µm particle size, analogous to B1) by slurry processing. The cells including a composite cathode made of NMC622, VGCF, and Oppanol as the binder showed stable cycling for 50 cycles.

## 4 Conclusion 

In this study the influence of the microstructure on the ionic conductivity and electrochemical properties of *β*‐LPS solid‐electrolyte pellets has been systematically investigated, proving that the secondary particle morphology, i.e., particle size and intrinsic porosity of single particles, is very crucial in determining the ionic conductivity and electrochemical performance at the Li metal interface. Although sulfides are considered to be very ductile and can be densified even at room temperature, the grain boundary resistance can represent a major hurdle. Herein, it is shown that the SE porosity and tortuosity have a minor impact on conductivity compared to grain boundaries. In fact, the fastest conduction is observed in pellets composed of particles with less grain boundaries, regardless of the degree of porosity. This new cognition may open new opportunities to rethink the design strategy of SSBs.

## Conflict of Interest

The authors declare no conflict of interest.

## Supporting information

Supporting information

## Data Availability

The data that support the findings of this study are available from the corresponding author upon reasonable request.
