# Supplementary material for: Elucidating the Role of Microstructure in Thiophosphate Electrolytes – a Combined Experimental and Theoretical Study of β‐Li3PS4
Source: Adv Sci (Weinh). 2022 Apr 24;9(18):2105234. doi: 10.1002/advs.202105234 (PMC9218768; doi:10.1002/advs.202105234)
Supplement: Supplementary file 1 — Supporting information [file ADVS-9-2105234-s001.pdf]

ADVANCED SCIENCE
Open Access

Supporting Information

for *Adv. Sci.*, DOI 10.1002/advs.202105234

Elucidating the Role of Microstructure in Thiophosphate Electrolytes – a Combined Experimental and Theoretical Study of β-Li_3PS_4

Tugce Ates, Anton Neumann*, Timo Danner*, Arnulf Latz*, Maider Zarrabeitia*, Dominik Stepien*, Alberto Varzi* and Stefano Passerini**

Elucidating the Role of Microstructure in Thiophosphate Electrolytes – a Combined Experimental and Theoretical Study of β-Li_3PS_4

Tugce Ates (tugce.ates@kit.edu)[a,b], Anton Neumann (anton.neumann@dlr.de)[a,c], Timo Danner (timo.danner@dlr.de)[a,c], Arnulf Latz (arnulf.latz@dlr.de)[a,c], Maider Zarrabeitia (maider.ipina@kit.edu)[a,b], Dominik Stepien (dominik.stepien@kit.edu)[a,b], Alberto Varzi (alberto.varzi@kit.edu)[a,b], Stefano Passerini (stefano.passerini@kit.edu)[a,b]

[a]*Helmholtz Institute Ulm (HIU), Helmholtzstrasse 11, 89081 Ulm, Germany*

[b]*Karlsruhe Institute of Technology (KIT), P.O. Box 3640, 76021 Karlsruhe, Germany*

[c]*German Aerospace Center (DLR), Institute of Engineering Thermodynamics Pfaenwaldring 38-40, 70569 Stuttgart, Germany*

Supplementary Information (SI)

Particle Characterization

Lattice Parameters

The calculated lattice parameters from XRD and the theoretical density of the different LPS batches are shown in *Table SI.1*. The values are in agreement, with literature values, see Ref.[1].

Table SI.1: Lattice parameters and theoretical densities calculated from XRD patterns:

	a **(Å)**	***b*** **(Å)**	***c*** **(Å)**	**Volume (Å³)**	**Density (gcm^{-3})**
**B1**	12.9793(8)	8.0405(5)	6.1352(4)	640.27(8)	**1.868**
**B2**	12.9839(7)	8.0414(4)	6.1327(3)	640.31(7)	**1.868**
**B3**	12.9821(10)	8.0455(6)	6.1335(4)	640.63(11)	**1.867**
**B4**	12.9825(10)	8.0448(7)	6.1344(5)	640.69(12)	**1.866**

XPS

To analyse the chemical surface composition of the different batches X-ray photoelectron spectroscopy (XPS) was used. The measurements were performed using a monochromatic Al Kα source (hν = 1486.6 eV) and a Phoibos 150 XPS spectrometer with a microchannel plate and a delay line detector (DLD) in fixed analyser transmission mode. High-resolution scans were collected at 400 W, 30 eV pass energy, and 0.1 eV energy step. The photoelectron spectra were calibrated using the hydrocarbons peak (-C-C-/-C-H-) at 284.8 eV as reference. The data evaluation was carried out with CASAXPS software using a nonlinear Shirley-type background and Voight profile function.

The spectra of the four β-LPS batches are shown in *Fig.SI.1* (from bottom to top: B1 to B4). For clarity, the Li 1s, P 2p, S 2p, C 1s and O 1s spectra are plotted separately (from left to right). In all samples the **S 2p** spectra features the S^{2-} doublet peaks ($2p_{1/2}$ and $2p_{2/3}$) of Li_3PS_4 at 161.5 eV and 162.5 eV, respectively.[2,3,4] Additionally a peak toward higher binding energies appears, which can be assigned to adsorbed H_2S[5] and appears particularly pronounced for B2 and B3. In fact, B2 and B3 show 2-3 times higher H_2S content (*3.1% and 3.9%)* compared to B1 and B4 (*1.1% and 1.4%*). On the other hand, B1 and B4 have accordingly higher content of pure Li_3PS_4 at the surface (*27.3% and 25.8%*). The **P 2p**

spectra are in accordance with the S 2p spectra, confirming the doublets appearing at 133.3 eV and 132.5 eV assigned to PS_4^{3-}.[2,3,4]

Besides the expected P and S compounds also C and O compounds could be detected on the electrolyte surface of all four batches. The surface of the SE contains products like *e.g.*, ethers (-COC-), alcohols(-COH), and carboxyl/esters (-COOH/-COOR) originating, most probably, from reaction with the atmosphere.[6] But having a closer look at the O 1s and Li 1s spectra of B2 and B3, lithium hydroxide, LiOH, could be detected, whereas the surface of B3 contains the highest amount on LiOH (*4.20%*).[7] As we know, that LPS is sensitive towards humidity[8], we can assume that B2 and B3 have been in contact with moisture triggering the formation of LiOH compounds on the surface, also in accordance with the increased content of H_2S for these SE batches.[9] Surface impurities play a crucial role on the interface formation with Li metal, though, later, we will see that the influence of the different surface impurities of the SE batches seem to have a not significant influence on the electrochemical performance.

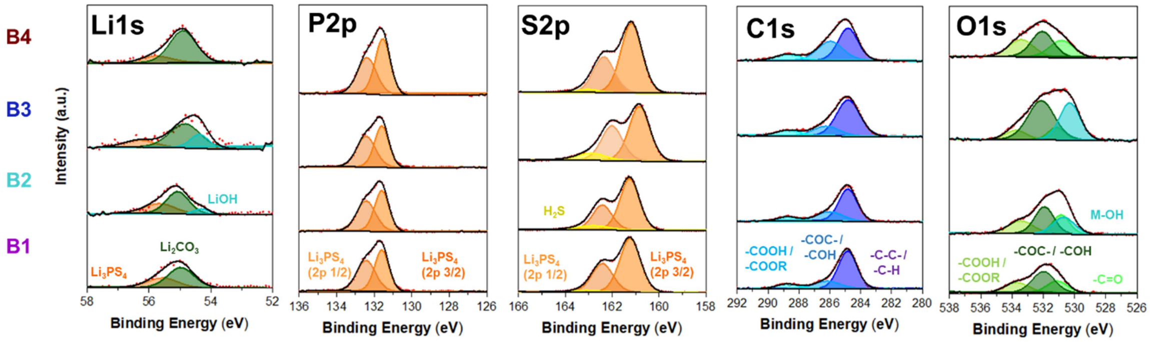

Figure SI.1: XPS photoelectron spectra of B1, B2, B3, and B4 (from bottom to top). Spectra of C 1s, O 1s, Li 1s, S 2p, P 2p (from left to right).

SEM – Particle Sizes

In *Fig.SI.2*, higher magnification SEM images of all 4 batches are shown. The particles size was determined by Image J. The secondary particles were considered for the given average particle sizes of 20.69 ± 3.81 µm for B1 (20 µm), 6.90 ± 1.67 µm for B2 (7 µm) and 3.71 ± 0.89 µm for B3 (3µm) and 5.53 ± 1.14 µm B4 (3 µm). B3 and B4 do not display the characteristic rod-like particle shape, the secondary particle sizes are therefore much smaller, and they tend to easily form agglomerates.

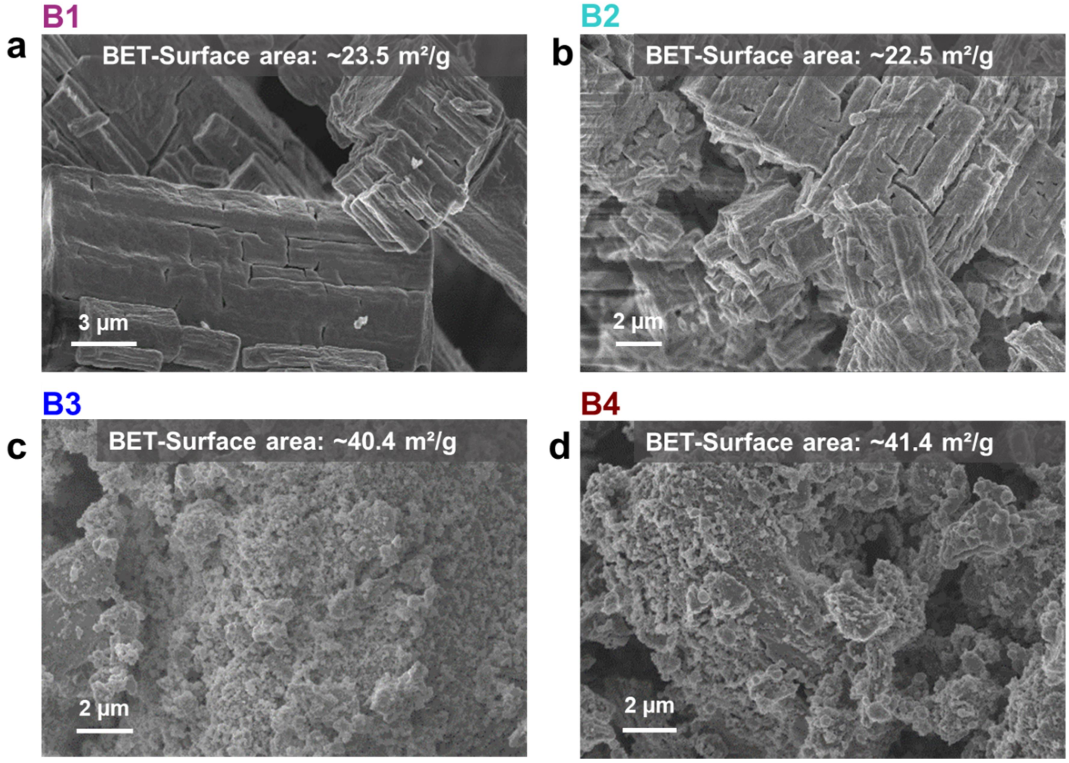

Figure SI.2: SEM images of a) B1, b) B2, c) B3 and d) B4.

Pellet Characterization

SEM

The cross-sectional images of a B1 pellet pressed under active vacuum are shown in *Fig.SI.3 a-b).* The comparison with Fig. 4a shows that no further densification was achieved.

A single LPS particle from B1 was then analysed further. The cross-sectional images shown in *Fig SI.3 c-d)* reveals the existence of a large porosity between single crystallites within the single particle. Such intrinsic porosity is, apparently, not removed by cold-pressing.

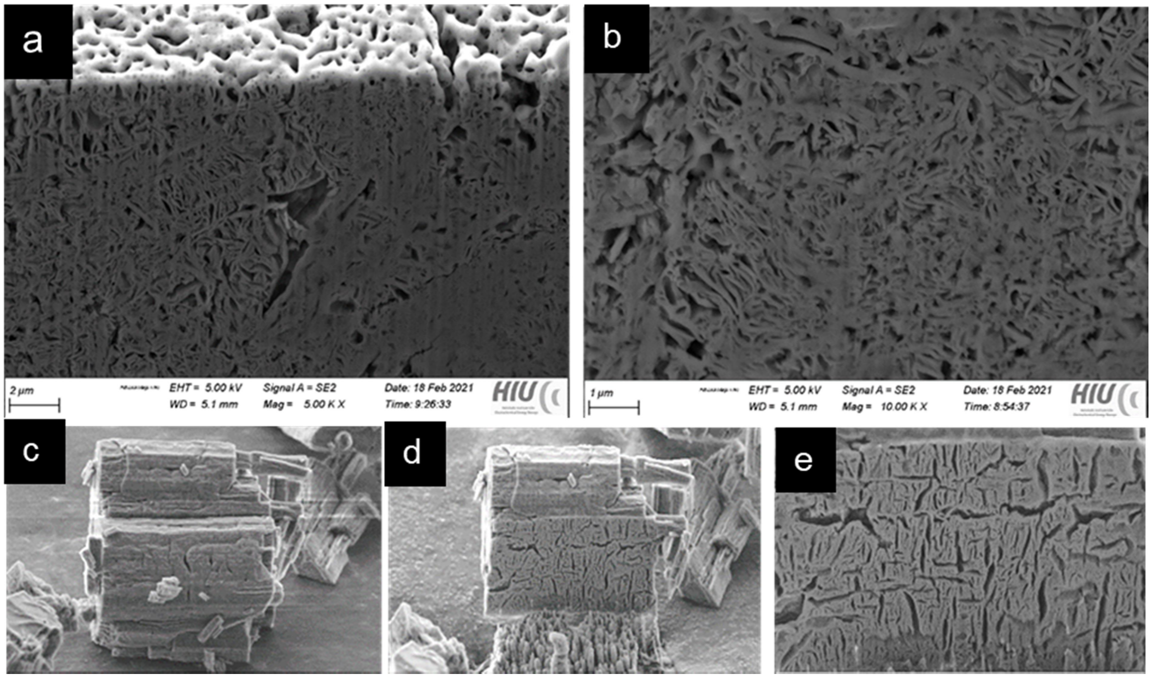

Figure SI.3: SEM images of a) B1 pellet pressed under active vacuum at low magnification and b) high magnification. SEM images of c) pristine B1 particle, d) cross section of a B1 particle at low magnification and e) at high magnification.

SAXS

The SAXS patterns were collected using a Xeuss 3.0c (Xenocs – Grenoble, France) equipped with an Eiger2 1M detector. The Sample-to-Detector distance was set to 1100 mm. A Mo Kα source was exploited with a beam size of 0.35x0.35 mm^2, obtaining a flux of ~10^5 photons per sec. The pellet samples where sticked on a perforated metal plate, but the optic path of the X-rays was not covered with tape. The sample chamber of the instrument was kept under vacuum (P=15 µbar) during the experiment. Each pellet sample was exposed for 20 minutes to X-rays to ensure a good signal-to-noise ratio. The tape background was also collected and subtracted from the total scattering curve. Data treatment was performed with the SAXSutilities2 software.

In *Fig.SI.4* the log-log plot of the SAXS curves are shown. All batches show polydisperse distribution of the pore shapes. B1 and B2 obtain oblate spherical pores, whereas B3 and B4 obtain two different slopes, resulting from two different pore shapes, oblates and disks.

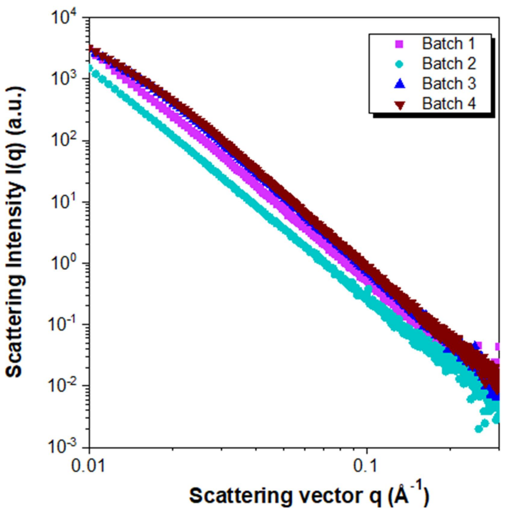

	Porosity shape	Average diameter
Batch1	oblate spheres	20.2 nm
Batch2	oblate spheres	51.6 nm
Batch3	disks + oblate spheres	17.9 nm
Batch4	disks + oblate spheres	15.2 nm

Figure SI.4: SAXS curve and calculated porosity shapes and average pore diameter (measured with Molybdenum source).

Particle size reduction study of sample B1

B1 SE was grinded in two different ways. One was manual grinding (MG) with a mortar and pistil for ca. 30 mins, the other way was soft ball milling (SB). For the SB method a centrifuge tube was used. 500 mg SE powder and different sizes of zirconia balls were added (5 balls of 1 cm diameter, 10 balls of 5 mm diameter + 20 balls of 2 mm diameter), afterwards the tube was placed overnight on a roller mixer with a speed of 60 rpm. In *Fig.SI.5 a)* the conductivity results of the pristine B1 batch and the results of the grinded samples are plotted. *In Fig.SI.5 b-c)* the SEM images of the particles grinded via MG and SB are shown, respectively.

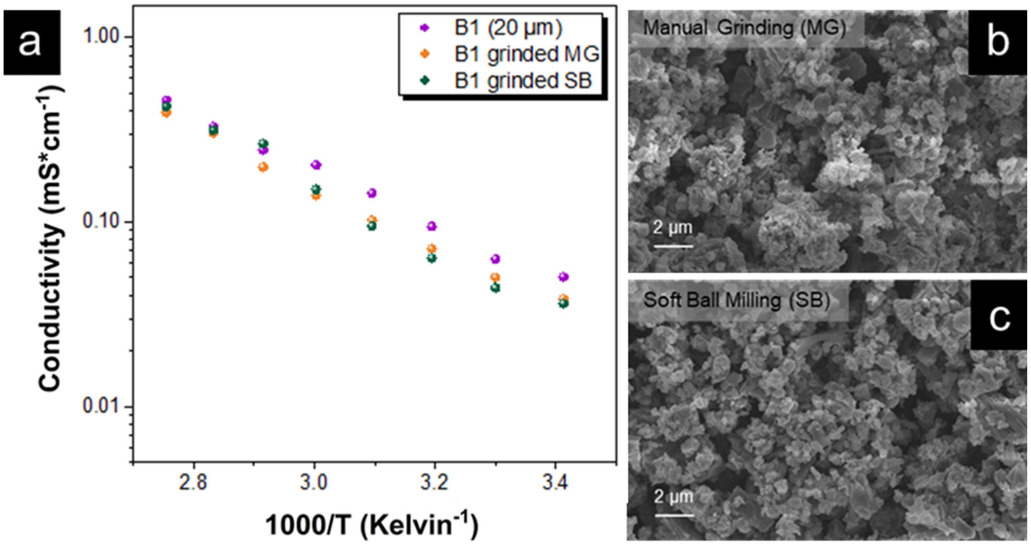

Figure SI.5: a) Ionic conductivity determined in symmetrical cells with stainless steel blocking electrodes. In violet: B1, in orange: B1 grinded by manual grinding and in green: B1 grinded by soft ball milling. SEM-images of the grinded B1 samples b) by manual grinding and c) by soft ball milling.

Microstructure Generation

The SEM analysis indicates that the particle morphology and size for the four LPS batches varies significantly. Notably, for B1 the estimated average particle diameter of 20 µm reduces to 7 µm for B2 and decreases for B3 and B4 further down to 4-5 µm. Another indication for the reducing particle size is the increased surface area measured via BET. The measurements show an increase by a factor of two between B1-B2 and B3-B4. The combination of both experimental results is taken as a starting point to investigate the influence of particle morphology on the pellet resistance. As shown in *Figure SI.3*, the particle surface is assumed to be sufficiently ductile and robust to allow a dense palletisation without reducing the internal particle fibrous structure.

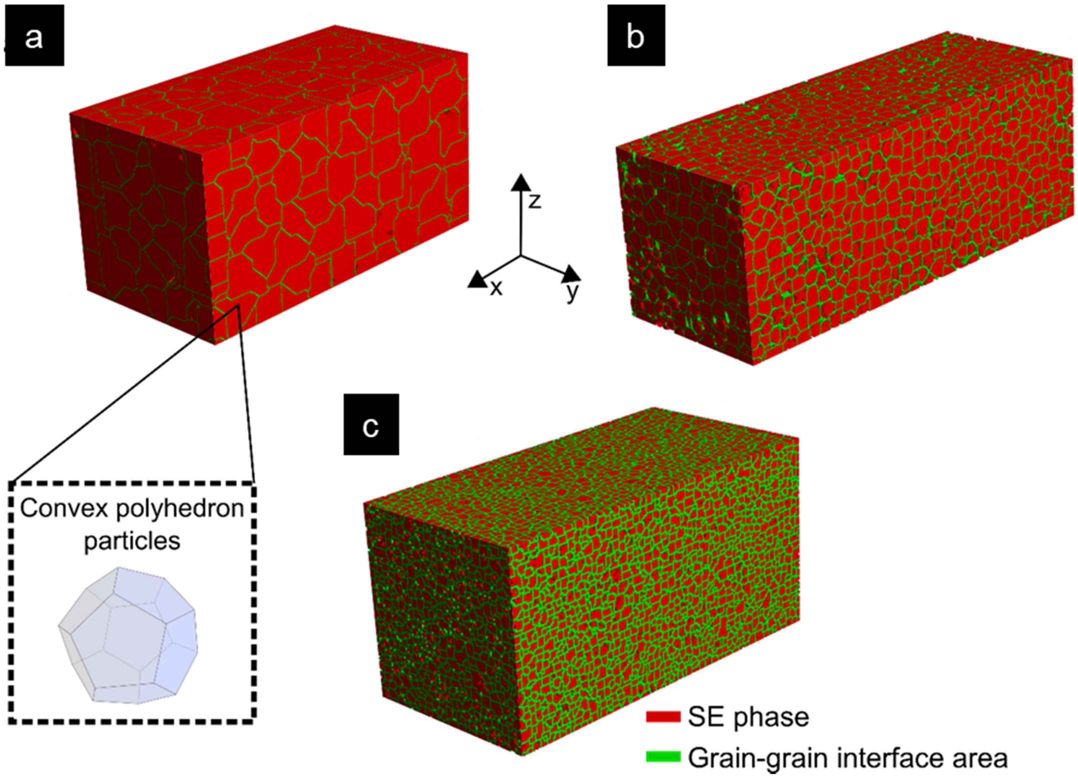

Figure SI.6: Segmented virtual microstructures with varying particle diameter: a) 20 µm particles, b) 7 µm particles and c) 3 µm particles. The bulk SE is shown in red and the grain-grain interface area introduced through the segmentation process is highlighted in green. For the later microstructure simulation, the GB interface flux model is defined at the segmentation lines between adjoint particles.

Three different SE microstructures were generated with varying particle diameter from 20 µm, 7 µm to 3 µm in agreement with the experiment. The microstructure generation is performed via GeoDict[10] and the respective grain segmentation step is later processed in Matlab. Starting with the GeoDict structure generator, we approximate the SE particles through a convex polyhedron shape and randomly place the particles in the simulation domain. The diameter of the randomly distributed particles is based on a gaussian distribution. Segmentation in individual grains is in a second step done using a watershed-based image processing routine in Matlab. The resulting segmented microstructures are shown in *Fig.SI.6 (a-c)*. Note, that the created SE samples have negligible residual porosity between grains and are fully densified. Further details on the image processing are given in Ref[11]

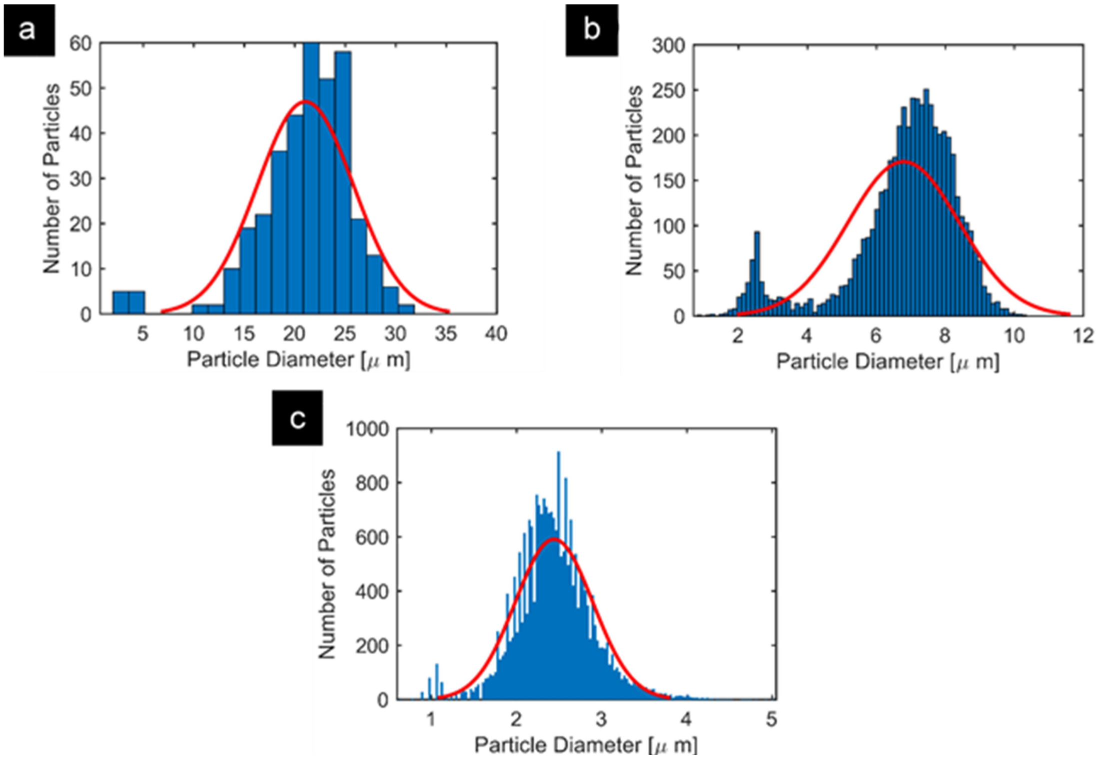

Figure SI.7: Particle size distribution after grain segmentation for the three different microstructures: a) 20 µm particles, b) 7µm particles and c) 3µm particles. The fitted Gaussian distribution is shown in red, and the calculated mean values and standard deviation are shown in the respective inset.

Particle Size Distribution

The particle size distribution is estimated during the segmentation step, at which the number of created particles and their respective voxel volume (V_P) is listed. The particle diameter, d_P, of a spherical particle is approximated with the same volume, which results in the following relation:

$$d_P = \frac{\left(\frac{6V_P}{\pi}\right)^{\frac{1}{3}}}{3} \qquad \textit{(Equation-SI.1)}$$

By applying *Eq-SI.1*, we can calculate the respective particle diameter.

Fig.SI.7 shows the grain diameter distribution for the structures with initial diameters of a) 20 µm, b) 7 µm, and c) 3 µm. Note that the Gaussian distribution fit for the mean value estimation is shown in solid red. As expected, the number of particles reduces with increasing particle diameter. Further structural parameters of the samples are given in *Table SI.1*. Note, that each LPS batch is assigned to a specific microstructure based on the SEM analysis. In particular, the 20 µm particle structure corresponds to B1, the 7 µm particle structure to B2

and the 3 μm particle structure to B3 and B4. Since the SEM images have a limited resolution and are only 2D, this assignment is not unique. Therefore, the obtained particle size distributions could potentially underestimate particle inhomogeneities or overestimate local effects.

Effective Bulk Conductivity Variation

The change in the effective bulk conductivity $\sigma_{eff,bulk}$ of the porous particles is calculated based on the variation in porosity (ε) and tortuosity (τ^2) by using the Bruggeman relation

$$\sigma_{eff,bulk} = \frac{\varepsilon}{\tau^2}\sigma_{bulk}. \qquad \text{(Equation-SI.2)}$$

The porosity is varied from 10% to 40% and the tortuosity from 1.3 to 2.0 to reproduce the experimentally measured particle properties. The resulting effective bulk conductivities calculated for the microstructure simulation study are given in *Table SI.2*.

Table SI.2: Calculated effective bulk conductivity values based on the Bruggeman relation to account for the particle porosity. The estimated conductivity values are given in units of [S/cm].

Effective bulk conductivity $\sigma_{eff,bulk}$				
Tortuosity τ^2	Porosity $\varepsilon = 0.6$	$\varepsilon = 0.7$	$\varepsilon = 0.8$	$\varepsilon = 0.9$
1.3	$9.23 \cdot 10^{-5}$	$1.08 \cdot 10^{-4}$	$1.23 \cdot 10^{-4}$	$1.38 \cdot 10^{-4}$
1.6	$7.50 \cdot 10^{-5}$	$8.75 \cdot 10^{-5}$	$1.00 \cdot 10^{-4}$	$1.13 \cdot 10^{-4}$
2.0	$6.00 \cdot 10^{-5}$	$7.00 \cdot 10^{-5}$	$8.00 \cdot 10^{-5}$	$9.00 \cdot 10^{-5}$

Microstructure Transport Modell BEST

A prominent advantage of the microstructure resolved simulations is the ability to incorporate inherently the morphological properties of materials and components in the battery cell, such as the particle size and electrode tortuosity. The battery simulation studies are performed on virtually generated voxel-based electrodes containing standard experimental microstructural parameters like pellet thickness and particle size. In *Table SI.3*, the relevant transport equations implemented in the framework BEST[12] are summarised:

Table SI.3: The constitutive equations of the Li-ion battery model used in this work. Indices are defined as solid electrolyte= SE, active material= AM.

Phase	Material balance	Charge balance
Electrolyte	$\frac{\partial \varepsilon_{\mathrm{El}} c_{\mathrm{El}}}{\partial t} = -\vec{\nabla} \cdot \vec{N}_{\mathrm{SE}}$ (1a)	$0 = -\vec{\nabla} \cdot \vec{j}_{\mathrm{SE}}$ (1b)
Active material	$\frac{\partial c_{\mathrm{So}}}{\partial t} = -\vec{\nabla} \cdot \vec{N}_{AM}$ (1c)	$0 = -\vec{\nabla} \cdot \vec{j}_{\mathrm{AM}}$ (1d)

Phase	Lithium flux	Charge flux
Electrolyte	$\vec{N}_{\mathrm{SE}} = \frac{\vec{j}_{SE}}{F}$ (1e)	$\vec{j}_{\mathrm{SE}} = -\sigma_{eff.,\ bulk}\vec{\nabla}(\varphi)$ (1f)
Active material	$\vec{N}_{\mathrm{AM}} = -D_{\mathrm{AM}}\vec{\nabla}c_{\mathrm{AM}}$ (1g)	$\vec{j}_{AM} = -\kappa_{AM}\vec{\nabla}\,\Phi_{AM}$ (1h)

Details on the modelling equations, the respective derivation, and further applications of the presented all-solid-state battery model are given in Ref.[11,13]. Specific performance and interface studies on thiophosphate-based composite cathodes can be found in Ref[14,15].

Grain Boundary Interface Flux

Different classes of polycrystalline SE, such as oxide-based materials, are well known to show pronounced limitation through the presence of grain boundaries. For thiophosphate-based SE, the direct imaging of grain boundaries is hindered through their low material stability under beam exposition in high-resolution imaging techniques such as Transmission-Electron Microscopy (TEM). Therefore, impedance analysis and general electrochemical characterization techniques are used to detect limiting factors, like GBs. For example, the observed differences in activation energy between NMR and EIS measurements on SE indicate the presence of additional transport limitations in the material. This could be residual porosity, amorphous interfaces or even GB contributions. Furthermore, symmetric lithium cell cycling of LPS provides an insight on the presence and influence of GBs. These studies indicate that dendrite formation is preferentially along particle boundaries. For our study, a distinct presence of GBs can only be assumed based on the SEM images of the different LPS batches. The resolved pellets cross-sections suggest that the particle morphology remains present in the compressed pellets. A schematic representation of the particle distribution is given in **Fig.1b)** of the main study, where each colour represents a single particle in the SE structure. This assumption justifies the application of a modified GB interface flux model to account for the additional GB resistance in the compressed SE.

Due to the high numerical cost of spatially resolving the nanometer scale GB phase at the grain-grain contact area inside the microstructure, the additional transport resistance is accounted by using a modified GB interface flux expression:

$$i_{GB} = k_{hop}\sqrt{\tilde{c}_{G_i}\tilde{c}_{G_J}}\left[exp\left(\frac{\Delta\tilde{\varphi}}{2RT}\right) - exp\left(-\frac{\Delta\tilde{\varphi}}{2R}\right)\right] \quad . \qquad \text{(Equation-SI.3)}$$

The grain-grain interface current is defined by the lithium hopping rate k_{hop} and the respective grain concentrations of lithium $\tilde{c}_{G_i}$. The driving force for the lithium hopping is given through the electrochemical potential difference $\Delta\tilde{\varphi}$ between the two individual grains. The resulting GB interface flux is used to describe the lithium hopping process at the grain boundary. The interface flux expression is defined at each grain interface of the segmented virtual SE structure. In **Fig.1a)**, we visualised the extend of the grain-grain interface area inside the segmented SE pellet.

Through this approach, we can account for resistive GB contributions at the grain-grain transition and enable microstructure resolved cell simulations at reasonable time scales. Note that the parameter k_{hop} is adjusted through experimental EIS measurements and therefore approximates the transport energy barrier for the lithium and additional morphological effects of the grain-grain contact. A detailed study of the presented model for an oxide-based porous SE network is given in Ref.[11] In the following, we further summarise the GB hopping rate (k_{hop}) and constant concentration contribution $\left(\sqrt{\tilde{c}_{G_i}\tilde{c}_{G_J}}\right)$ through the GB exchange current density $i_{00}^{GB} = k_{hop}\sqrt{\tilde{c}_{G_i}\tilde{c}_{G_J}}$.

The parametrisation of the GB interface flux model is based on the impedance measurement of LPS B1, since the high conductivity and the particle structure indicates the lowest influence of the GB resistance. We keep the GB interface parametrisation fixed for the three studied SE microstructures.

Model Parameterization

The following *Table SI.4* summarises the transport parameters and thermodynamic parameters of the simulated battery cell. The estimation of the transport parameters of β-LPS is based on the deconvolution of corresponding electrochemical impedance measurements of symmetric cells under blocking conditions. A detailed discussion can be found in Ref.[11]. The

general material parameters such as diffusion and conductivity were either measured or taken from literature.

Table SI.4: Simulation Parameters in BEST. Values are taken from the respective reference (superscript [Publication]), measured by the authors (superscript [°]), or calculated (superscript [*]). All other parameters are constant during the simulations.

Parameter, Unit	Description	Value
**Solid Electrolyte**		
$c_{Li}^{SE,0}$, mol/cm³	concentration of Li ions in ß-LPS *	0.0103
σ_{bulk}^{SE}, S/cm	Bulk Li-ion conductivity[16,17] .	$2.2 \cdot 10^{-4}$
$\sigma_{eff,bulk}^{SE}$, S/cm	Effective Li-ion conductivity *	Based on Bruggeman, see *Table SI.2*
t_{Li}^{+}	transference number of Li ions	1
D_{bulk}^{SE}, cm²/s	Li-ion self diffusion coefficient°	$2.4 \cdot 10^{-9}$
f_{Li}^{SE}	thermodynamic correction factor	1
LPS, µm	SE sample thickness °	See **Table 1**
**Grain Boundary**		
$i_{00}^{GB}, \mathrm{A/cm^2}$	exchange current density factor *	$8.24 \cdot 10^{-4}$
C_{DL}^{GB}, F/cm²	double layer capacity *	$1.1 \cdot 10^{-8}$
**Electrodes: conductivity analysis "No-resistance (GS)"**		
c_{Li}^{0}, mol/cm³	initial Li concentration *	0.0328
κ_{Li}, S/cm	electronic conductivity	100
D_{Li}^{GS}, cm²/s	Li-ion chemical diffusion coefficient	1
α^{GS}	symmetry factor	0.5
$i_{00}, \mathrm{A/cm^2}$	exchange current density factor *	2.0×10^{8}
$I_{ext.}$, mA/cm²	Applied external current	0.5
**Electrodes: impedance analysis "Blocking Configuration (BC) "**		
κ_{Li}^{BC}, S/cm	electronic conductivity *	100
D_{Li}^{BC}, cm²/s	Li-ion chemical diffusion	1

	coefficient	
α^{NMC}	symmetry factor	0.5
i_{00}^{BC}, A/cm²	exchange current density factor *	2.0×10^{-8}
C_{DL}^{BC}, F/cm²	double layer capacity *	$2.4 \cdot 10^{-5}$

Half Cell Analysis

EIS

EIS: Blocking Configuration

To determine the ionic conductivity of the four β-LPS batches and to analyse their interface with the lithium metal electrode, electrochemical impedance analysis was conducted. To determine the ionic conductivity, for each temperature, the R_{g+gb} was calculated by fitting the EIS curves with an equivalent circuit consisting of *(R(1))–(R(2)C(2))–(CPE)*, whereas R_1 was considered as R_{grain} (grain) and R_2 is considered as R_{gb} (grain boundary). Further, C is the capacitance and CPE the constant phase element. Some representative EIS curves @20°C are shown in *Fig.SI.8)*.

As both, R_{grain} and R_{gb} have an influence on the ionic conductivity, the sum of both values is used to calculate the ionic conductivities. By inserting the resulting R_{g+gb} values into the *equation-SI.4*, the ionic conductivities at each temperature are calculated.

$$\sigma = \frac{d}{S}\frac{1}{R_{g+gb}} \qquad \textit{(Equation-SI.4)}$$

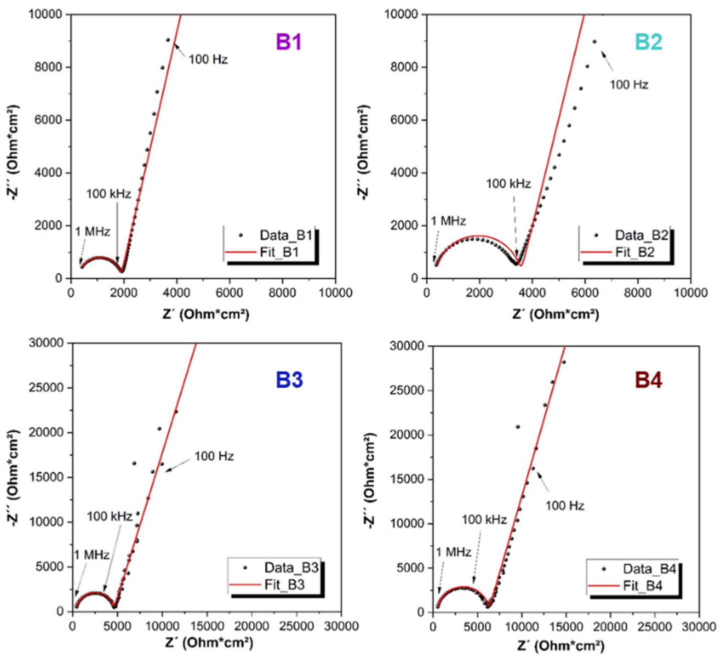

Figure SI.8: EIS Spectra and corresponding fit of symmetrical cells with stainless steel electrodes for ionic conductivity measurements. All measurements were conducted @20°C. In violet: B1, in turquoise: B2, in dark blue: B3 and in dark red: B4.

Where σ ionic conductivity, d = thickness of the SE pellet, S = area of electrodes contacting SE pellet, and R_{g+gb} = grain+ grain boundary resistance. The obtained resistances for grain and GB are given in *Table SI.5*.

Table SI.5: Calculated mean and standard deviation values for the grain $R_{mean,g}$, grain boundary $R_{mean,gb}$ resistance and the experimental grain conductivity $\boldsymbol{\sigma_{mean,g}}$. Note that $\boldsymbol{\sigma_{mean,g}}$ is equivalent to the utilized effective bulk conductivity values from Table SI.2. Thus, the fitted resistance partly accounts for the structurerelated bulk transport effects such as tortuosity and porosity.

**Batch**	$\boldsymbol{R_{mean,g}}$ $[\boldsymbol{\Omega}]$	$\boldsymbol{R_{mean,gb}}[\boldsymbol{\Omega}]$	$\boldsymbol{\sigma_{mean,g}} \times \boldsymbol{10^{-4}} \left[\frac{\mathbf{S}}{\mathbf{cm}}\right]$	$\frac{\boldsymbol{R_{mean,g}}}{\boldsymbol{R_{mean,gb}}}$
B1	382 ± 50	1979 ± 99	3.02 ± 0.35	0.19
B2	321 ± 64	3241 ± 162	3.99 ± 0.67	0.10
B3	407 ± 52	5040 ± 252	2.20 ± 0.25	0.08
B4	448 ± 58	5447 ± 272	1.71 ± 0.20	0.082

The resistance mean value and standard deviation calculation is based on four separate cells/ measurements. The relative ratio between R_g and R_{gb} is taken as an indicator for the dominating transport process. We observe that the ratio constantly decreases from 0.19 to 0.10 down to 0.08 for LPS B3 and B4. The ratio is a measure for the relative increase in GB resistance as the particle size decreases. This trend is in line with our assumption that the

conductivity reduction is mainly dominated by the increase in GB resistance contribution with reduced LPS particle size. Furthermore, we estimate the interface resistance per GB based on a geometric approximation of the LPS sample with varying particle sizes. The single interface GB resistance is approximated as:

$$R_{gb,int} = \frac{R_{mean,gb} \cdot A_{sampl}}{N_{gb}} \quad \text{(Equation-SI.5)}$$

with $N_{gb} \approx 2 \cdot \frac{L_{sample}}{d_{particle}}$.

Using a similar approach, we calculate our microstructure model's respective GB interface resistance. The simulated GB resistance $R_{gb,sim}$ is calculated based on the effective conductivity values from *Table SI.2* as:

$$R_{gb,sim} = R_{tot,sim} - R_{eff,bulk} = R_{tot,sim} - \frac{1}{\sigma_{eff,bulk}} \cdot L_{sample} \quad \text{(Equation-SI.6)}$$

Note that we selected the effective conductivity values from *Table-SI.2*. To simplify the comparison with the simulation results, a single conductivity value was selected, representing the experimental batch porosity for an intermediate tortuosity value of $\tau^2 = 1.6$. The obtained GB interface resistance values are shown in *Table SI.6*.

Table SI.6: Comparison of the interface resistance per GB taken obtained from the experimental and simulated data. The approximated particle diameter is provided for each LPS batch.

**Batch**	$R_{gb,int,exp}$ **[Ωcm]**	$R_{gb,int,sim}$ **[Ωcm]**	SE Particle Diameter
B1	17 ± 1	16	20µm
B2	9 ± 1	12	7µm
B3	11 ± 0.5	10	3µm
B4	14 ± 0.5		

The comparison between the simulated and experimental values shows a good agreement and indicates that the model parameters are able to represent the system at hand. Most importantly, we see that the GB interface resistance values are relatively constant over all four batches. Therefore, we can assume that the GB composition is not significantly changing between the various batches.

By using the Arrhenius equation (*Eq-SI.7*), the activation energies are calculated from the plotted conductivities.

$$\sigma = \frac{A}{T} n \, exp\left(-\frac{E_a}{k_b T}\right) \qquad \textit{(Equation-SI.7)}$$

Whereas σ is the ionic conductivity, T is temperature, A is a pre-exponential factor, n is the concentration of mobile-ion carriers, E_a is the activation energy of thermally activated process and k_b is the Boltzmann constant.

The calculated values for the ionic conductivities @20°C and the values for the activation energies are presented in *Table SI.7*.

Table SI.7: Ionic conductivities and activation energies for β-LPS batches B1, B2, B3 and B4 at 20°C for cells with stainless steel electrodes.

	**Ionic conductivity @20°C (Scm^{-1})**	**Activation energy (eV)**
**B1**	$4.93 \times 10^{-5} \pm 0.57 \times 10^{-5}$	0.29 ± 0.02
**B2**	$3.67 \times 10^{-5} \pm 1.70 \times 10^{-5}$	0.31 ± 0.01
**B3**	$1.57 \times 10^{-5} \pm 0.16 \times 10^{-5}$	0.33 ± 0.02
**B4**	$1.51 \times 10^{-5} \pm 0.59 \times 10^{-5}$	0.32 ± 0.02

EIS: Symmetric Lithium Cell

The EIS Spectra for symmetrical lithium cells are shown in *Fig.SI.9*.

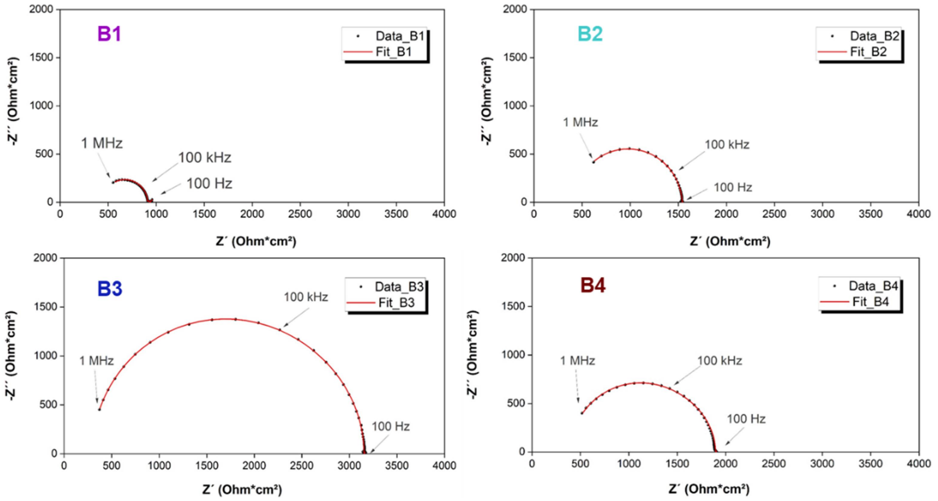

Figure SI.9: EIS Spectra and corresponding fit of symmetrical cells with lithium for stripping/plating. All measurements were conducted @20°C. In violet: B1, in turquoise: B2, in dark blue: B3 and in dark red: B4.

The applied equivalent circuit in this case consists of $(R_{(1)})$-$(R_{(2)}C_{(2)})$-$(R_{(3)}C_{(3)})$-W. Whereas R_1 is representative as R_g, R_2 is considered as R_{gb} and R_3 is standing for R_{int} (interfacial resistance), which is determined by R_{CT} (Charge Transfer) and R_{SEI} (Solid Electrolyte interface). Whereas W is standing for Warburg element. During the aging experiment shown in **Fig.7**, the interface resistance R_{int} is evolving overtime due to reactivity between LPS and the Li metal. The R and C values for the day 1 of aging are reported in *Table.SI.8*.

Table SI.8: The Resistance and Capacitance values from the fitting.

**Ageing Test Day1 (20°C)**	R_1 (Ohm)	R_2 (Ohm)	C_2 (F)	R_3 (Ohm)	C_3 (F)
**B1**	563	602	4.2×10^{-10}	31	3.2×10^{-6}
**B2**	544	1416	2.3×10^{-10}	15	2.3×10^{-7}
**B3**	380	3130	2.7×10^{-10}	529	3.3×10^{-9}
**B4**	511	1669	2.9×10^{-10}	250	5.7×10^{-9}

An additional experiment was performed with the SE B4. Li/Li symmetric cells were assembled with different amounts of SE (thus different thicknesses). The *Fig.SI.10* shows that the semicircle is related mostly to the grain-boundary resistance R_2.

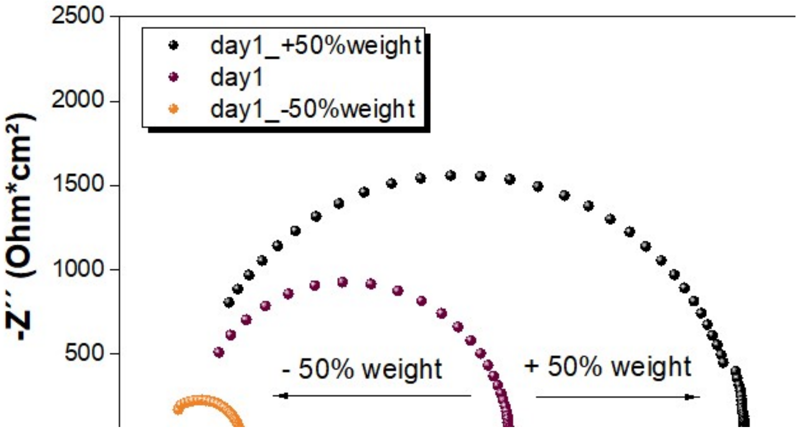

Figure SI.10: Nyquist plot of B4 in Li/Li symmetrical cells, with different amount of SE. All measurements were conducted @20°C. In orange: 75 mg of B4, in dark red: 150 mg of B4 and in black: 225 mg of B4.

Microstructure Impedance Response

The impedance simulation setup is adapted from the experimental lab system using steel stamps to create blocking electrode conditions. The interface parametrisation of the virtual blocking steel electrode is adjusted to reproduce the experimentally observed low frequency impedance tail. The respective parameters for the impedance parameterisation in this study are listed in *Table SI.4*. Details on the impedance simulations and the interface model definition are given in Ref.[18,11]

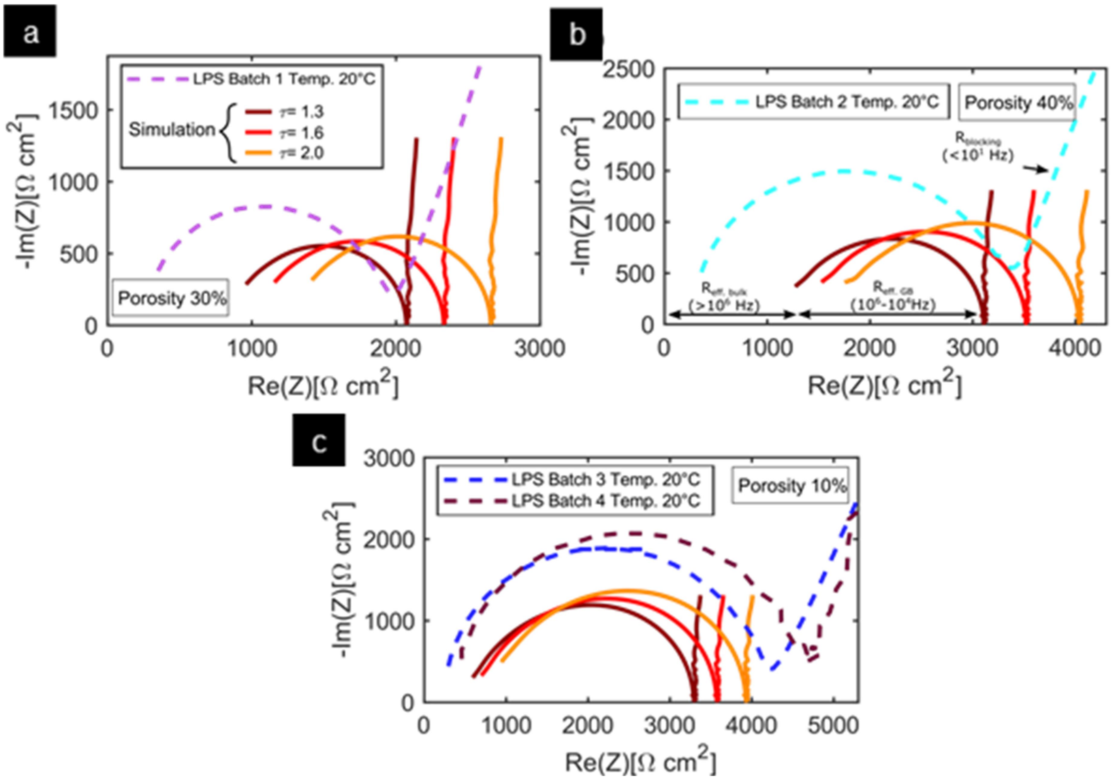

Figure SI.11: Nyquist plot representation of the experimental and simulated impedance response for the three different SE microstructures: a) LPS batch 1 (32% porosity) and 20 µm particle microstructure, b) LPS batch 2 (42% porosity) and 7 µm particle microstructure and c) LPS batch 3 and 4 (5% and 10% porosity) and 3 µm particle microstructure. The dotted lines are showing the experimental values and the simulated impedances are displayed in solid lines. The red colour map gives the variation in tortuosity from 1.3 to 2.0 and the simulated porosity is given by the respective inset.

Fig.SI.11 shows the Nyquist plot for the measured impedance of the four LPS batches with steel stamps at 20°C (dotted lines). Moreover, we added the simulated impedance responses at varying particle tortuosity for a fixed particle porosity (straight lines). The virtual porosity value is set to reproduce the experimental pellet porosity (cf. **Table 1**). The variation of the tortuosity τ for fixed porosity visualises the qualitative influence on the impedance (colour code). Nevertheless, the agreement between the theoretical and experimental impedance

response and the respective conductivity values indicates that the suggested virtual microstructures provide a qualitative representation of the pellet structure.

The simulated impedance response accounts for the GB resistance contribution using the interface flux model. As discussed in the main article, we see that the GB contribution increases with the specific particle surface area for the different structures (cf. *Table SI.9*). The high-frequency bulk contribution is currently not described in our model, but the respective resistance contribution and the resulting effective bulk conductivity is given through the constant shift along the real x-axis. Since the resistance contribution depends on the effective bulk conductivity, the shift along the x-axis is changing significantly under variation of the tortuosity and porosity. The GB contribution lies in the high to mid-frequency regime and is given by the fully resolved semi-circle. Additionally, the increasing particle tortuosity also leads to an observable rise in the GB polarisation. In the low frequency regime, the blocking tail behaviour is reproduced. The respective regimes are indicated in *Fig.SI.11b.* Furthermore, we provided frequency dependent data in Fig. for simulations and measurements on LPS batch 1 data from *Fig.SI.11a.*

It should be noted that in the experimental setup we assume that contributions of the bulk polarization are overlapping with the GB contributions. Therefore the deconvolution of both processes is not straightforward. Thus, the microstructure GB model allows us to selectively model the GB polarization and compare this to the measurements. This provides us with qualitative insight on the relation between the GB resistance, the particle morphology and the GB transport kinetics. For a more detailed description of the utilized EIS modelling approach on solid electrolytes and the respective frequency-dependent effects on the GB transport, we refer to Ref.[11].

We see that the simulated impedance response for B1 and B2 shown in **Fig.3a** and **Fig.3b** reproduces the experimental impedance data. Nevertheless, we observe a mismatch in the bulk contribution for B3 and B4 between simulation and experiment. We assume that the selected ionic bulk conductivity in our simulation overestimates the experimental value of B3 and B4, although compared to B1 and B2, the effective conductivity value is higher due to the reduced porosity and tortuosity (see *Table SI.2*). The mismatch might indicate an additional change in the bulk conductivity for B3 and B4 with decreasing particle size.

Since we use a fixed bulk conductivity value ($\sigma_{bulk} = 2.2 \cdot 10^{-4}$ S cm^{-1}) modified by the Bruggeman relation (Equation 2), we do not account for potential bulk conductivity changes

which might result from degradation during the assumed milling step of the material or are due to a smaller crystallite size. As shown via SEM analysis, the internal particle porosity is reduced in B3 and B4, which potentially reduces conduction pathways along the surfaces of the material. This interpretation is in line with our observation that the larger particles in B1/B2 have smoother particle surfaces and show less particle agglomeration. The observed particle morphology trend would suggest that the transport along and inside the larger and smoother particles is improved compared to the batches with smaller particles showing stronger surface roughness and agglomeration[19].

We performed an additional parameter study to investigate the influence of bulk conductivity on the impedance response of B3/B4. Therefore, we selected the 3µm pellet structure and varied the bulk conductivity value from $2.2 \cdot 10^{-4}$ S cm^{-1}, $1.5 \cdot 10^{-4}$ S cm^{-1} to $1.0 \cdot 10^{-4}$ S cm^{-1} with a fixed tortuosity ($\tau^2 = 1.3$) and porosity ($\varepsilon = 0.9$). The selected bulk values are based on the experimentally measured LPS conductivities found in the literature [16,17,19]. The simulated impedance response (solid line) and the experimental measurement (dotted line) are shown in *Fig.SI.12*.

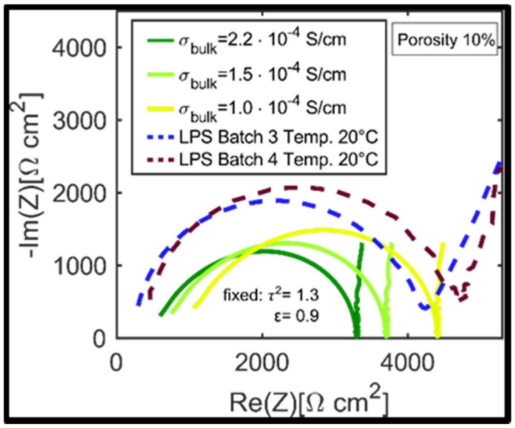

Figure SI.12: Nyquist plot representation of the simulated impedance response for the 3µm particle microstructure for three different bulk conductivity values σ_{bulk}. The dotted lines are showing the experimental values, and the simulated impedances are displayed in solid lines. The red colour map gives the variation in the bulk conductivity. The inset gives the simulated porosity and tortuosity values.

The change in bulk conductivity leads to the assumed shift in the bulk contribution. Again, the decreasing conductivity gives rise to an increase in GB contribution, which results from the fixed GB parameter set we used for all simulation studies. Nevertheless, the agreement between simulation and experiment for B3 and B4 is improved through the reduced bulk conductivity value. We conclude that additional mechanisms might cause the decrease in

bulk conductivity between batches B1/B2 and B3/B4. This effect would be in line with the experimental findings. Although batches B1/B2 possesses a higher porosity than B3/B4, the decreased particle size and higher GB contribution and the reduced bulk conductivity lead to decreased effective pellet conductivity. Further experimental and theoretical investigations are necessary to verify our assumption of the decreasing bulk conductivity with decreasing particle size, which is beyond the scope of this study.

We conclude that the presented impedance study provides an additional analysis method to investigate the structural aspects of the SE, such as porosity and particle size, and crosslink them to electrochemical transport phenomena at GB and limit interface phenomena. Furthermore, since the cell performance is significantly impacted by surface composition and particle morphology, future cell studies will further investigate the aspects.

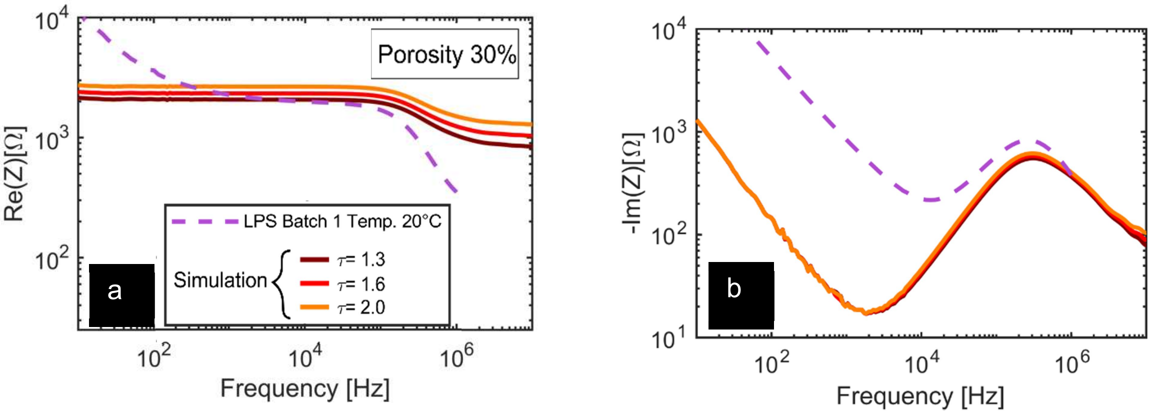

Figure SI.13 Frequency dependent representation of the a) real and b) imaginary resistance contribution of the experimental and simulated impedance response: LPS batch 1 (32% porosity) and 20 µm particle microstructure. The dotted lines are showing the experimental values and the simulated impedances are displayed in solid lines. The red coulor map gives the variation in tortuosity from 1.3 to 2.0 and the simulated porosity is given by the respective inset.

Frequency-Dependent Impedance Representation

Fig.SI.13 shows the Bode plot representation for the experimental impedance response for LPS batch 1 and the respective simulation with a fixed particle porosity and variation of the internal particle tortuosity. The comparison shows that the high- to mid-frequency regime (10^6-10^4 Hz) is captured qualitatively through the GB model. In the simulation the high-

frequency polarization is partly represented through the constant shift along the y-axis accounting for the effective bulk resistance resulting from tortuosity, porosity and general sample constriction. The observed ideal blocking-tail behaviour in the simulation is shifted towards lower-frequencies due to the assumed perfect contact between SE and current collector.[20] Note that we do not include any specific contact surface modification and therefore can only provide a qualitative description of the capacitive behaviour at low frequencies in the simulations. .

Specific Particle Surface Area

Table SI.9: Calculated structural parameters for the virtual microstructures: average particle diameter and particle specific surface area. The respective particle distributions are given in the supporting information.

**Parameter, Unit**	**Structure 20**	**Structure 7**	**Structure 3**
Average particle diameter, μm	20	7	3
Particle specific surface area, × $\mathbf{10^3 cm^{-1}}$	3.8	8.0	15.8

REFERENCES:

1. Homma, K., Yonemura, M., Nagao, M., Hirayama, M. & Kanno, R. Crystal structure of high-temperature phase of lithium ionic conductor, Li3PS4. *J. Phys. Soc. Japan* **79,** 90–93 (2010).

2. Kato, A., Kowada, H., Deguchi, M., Hotehama, C. & Hayashi, A. XPS and SEM analysis between Li / Li 3 PS 4 interface with Au thin fi lm for all- solid-state lithium batteries. *Solid State Ionics* **322,** 1–4 (2018).

3. Liang, J. *et al.* In Situ Li3PS4 Solid-State Electrolyte Protection Layers for Superior Long-Life and High-Rate Lithium-Metal Anodes. *Adv. Mater.* **30,** (2018).

4. Lu, Y. *et al.* Pre-modified Li 3 PS 4 based interphase for lithium anode towards high-performance Li-S battery. *Energy Storage Mater.* **11,** 16–23 (2018).

5. Leavitt, A. J. & Beebe, T. P. Chemical reactivity studies of hydrogen sulfide on Au(111). *Surf. Sci.* **314,** 23–33 (1994).

6. Blyth, R. I. R. *et al.* XPS studies of graphite electrode materials for lithium ion

batteries. *Appl. Surf. Sci.* **167,** 99–106 (2000).

7. Wood, K. N. & Teeter, G. XPS on Li-Battery-Related Compounds: Analysis of Inorganic SEI Phases and a Methodology for Charge Correction. *ACS Appl. Energy Mater.* **1,** 4493–4504 (2018).

8. Muramatsu, H., Hayashi, A., Ohtomo, T., Hama, S. & Tatsumisago, M. Structural change of Li2S-P2S5 sulfide solid electrolytes in the atmosphere. *Solid State Ionics* **182,** 116–119 (2011).

9. Zhu, Y. & Mo, Y. Materials Design Principles for Air-Stable Lithium/Sodium Solid Electrolytes. *Angew. Chemie - Int. Ed.* **59,** 17472–17476 (2020).

10. GeoDict by Math2Market, commercial software for structure analysis and generation; https://www.math2market.com/?Language=de; Accessed: 2019-09-18.

11. Neumann, A. *et al.* Effect of the 3D Structure and Grain Boundaries on Lithium Transport in Garnet Solid Electrolytes. *ACS Appl. Energy Mater.* **4,** 4786–4804 (2021).

12. Latz, A. & Zausch, J. Multiscale modeling of lithium ion batteries: Thermal aspects. *Beilstein J. Nanotechnol.* **6,** 987–1007 (2015).

13. Finsterbusch, M. *et al.* High Capacity Garnet-Based All-Solid-State Lithium Batteries: Fabrication and 3D-Microstructure Resolved Modeling. *ACS Appl. Mater. Interfaces* **10,** 22329–22339 (2018).

14. Neumann, A. *et al.* Analysis of Interfacial E ff ects in All-Solid-State Batteries with Thiophosphate Solid Electrolytes. *ACS Appl. Mater. Interfaces* **12,** 9277–9291 (2020).

15. Randau, S. *et al.* On the Additive Microstructure in Composite Cathodes and Alumina-Coated Carbon Microwires for Improved All-Solid-State Batteries. *Chem. Mater.* **33,** 1380–1393 (2021).

16. Stöffler, H. *et al.* Amorphous versus Crystalline Li3PS4: Local Structural Changes during Synthesis and Li Ion Mobility. *J. Phys. Chem. C.* **123,** 10280–10290 (2019).

17. Stöffler, H. *et al.* Li + -Ion Dynamics in β-Li 3 PS 4 Observed by NMR: Local Hopping and Long-Range Transport. *J. Phys. Chem. C* **122,** 15954–15965 (2018).

18. Hein, S. *et al.* Influence of Conductive Additives and Binder on the Impedance of Lithium-Ion Battery Electrodes: Effect of Morphology. *J. Electrochem. Soc.* **167,**

13546 (2020).

19. Liu, Z. *et al.* Anomalous high ionic conductivity of nanoporous β - Li 3 PS 4. *J. Am. Chem. Soc.* **135,** 975–978 (2013).

20. Parejiya, A. *et al.* Improving Contact Impedance via Electrochemical Pulses Applied to Lithium-Solid Electrolyte Interface in Solid-State Batteries. *ACS Energy Lett.* **6,** 3669–3675 (2021).
